# Mechanistic convergence of the TIGIT and PD-1 inhibitory pathways necessitates co-blockade to optimize anti-tumor CD8^+^ T cell responses

**DOI:** 10.1016/j.immuni.2022.02.005

**Published:** 2022-03-08

**Authors:** Karl L. Banta, Xiaozheng Xu, Avantika S. Chitre, Amelia Au-Yeung, Chikara Takahashi, William E. O’Gorman, Thomas D. Wu, Stephanie Mittman, Rafael Cubas, Laetitia Comps-Agrar, Amit Fulzele, Eric J. Bennett, Jane L. Grogan, Enfu Hui, Eugene Y. Chiang, Ira Mellman

**Affiliations:** 1 Genentech, Inc., 1 DNA Way, South San Francisco, CA 94080, USA; 2 Section of Cell & Developmental Biology, Division of Biological Sciences, University of California, San Diego, 9500 Gilman Drive, La Jolla, CA 92093, USA; 3 Present address: Iovance, 3802 Spectrum Blvd., Tampa, FL 33612, USA; 4 Present address: Graphite Bio, 279 E. Grand Avenue, South San Francisco, CA 94080, USA; 5 These authors contributed equally; 6 Senior author; 7 Lead contact

## Abstract

Dual blockade of the PD-1 and TIGIT coinhibitory receptors on T cells shows promising early results in cancer patients. Here, we studied the mechanisms whereby PD-1 and/or TIGIT blockade modulate anti-tumor CD8^+^ T cells. Although PD-1 and TIGIT are thought to regulate different costimulatory receptors (CD28 and CD226), effectiveness of PD-1 or TIGIT inhibition in preclinical tumor models was reduced in the absence of CD226. CD226 expression associated with clinical benefit in patients with non-small cell lung carcinoma (NSCLC) treated with anti-PD-L1 antibody atezolizumab. CD226 and CD28 were co-expressed on NSCLC infiltrating CD8^+^ T cells poised for expansion. Mechanistically, PD-1 inhibited phosphorylation of both CD226 and CD28 via its ITIM-containing intracellular domain (ICD); TIGIT’s ICD was dispensable, with TIGIT restricting CD226 co-stimulation by blocking interaction with their common ligand PVR (CD155). Thus, full restoration of CD226 signaling, and optimal anti-tumor CD8^+^ T cell responses, requires blockade of TIGIT and PD-1, providing a mechanistic rationale for combinatorial targeting in the clinic.

## In brief

Dual blockade of the PD-1 and TIGIT coinhibitory receptors on T cells shows promising early results in cancer patients. Banta et al. find that PD-1 and TIGIT disrupt activation of the costimulatory receptor CD226 through distinct mechanisms, providing mechanistic rationale for the dual blockade of PD-(L)1 and TIGIT in cancer immunotherapy.

## Graphical Abstract

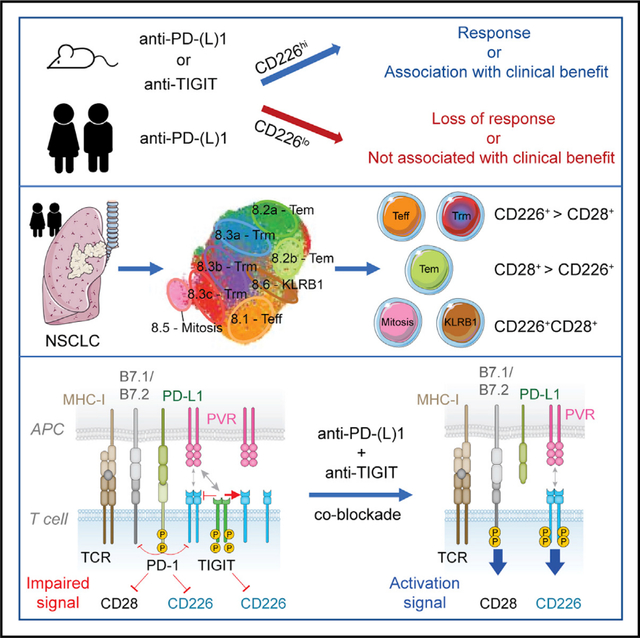

## INTRODUCTION

T cell inhibitory receptors, such as CTLA-4, PD-1, and TIGIT, are essential for limiting immunopathology and terminating effective immune responses, but also can restrain effective anti-tumor immune responses. Immunotherapeutic antibodies directed against these inhibitory receptors, including ipilimumab, nivolumab, pembrolizumab, atezolizumab, and tiragolumab, aim to enhance and reinvigorate antigen-specific T cell effector responses and have elicited clear or promising activity in cancer patients (Chen and Mellman, 2013). Although durable, responses have been limited to a minority of patients, creating considerable interest in exploring combinations of these antibodies with each other or with other therapeutic agents. Given the emerging complexities with regards to cell type expression, regulation, and function of PD-1, CTLA-4, and TIGIT (Yost et al., 2021), a greater understanding of the mechanisms associated with each may provide clues as to which combinations will be most effective.

Each coinhibitory receptor acts to regulate the activity of an important costimulatory receptor. CTLA-4 is well known to counteract CD28, and more recently, CD28 was also shown to be regulated by PD-1 (Hui et al., 2017). Indeed, inhibition of CD28 abolishes the ability of CD8^+^ T cells to respond to PD-1 blockade *in vivo* (Kamphorst et al., 2017). Although less well studied, CD226 is a costimulatory receptor regulated by TIGIT and exhibits multifaceted functions in anti-tumor CD8^+^ T cell responses (Gilfillan et al., 2008; Manes and Pober, 2011; Ralston et al., 2004; Shibuya et al., 1999, 2003; Shirakawa et al., 2005). The importance of CD226 in regulating anti-tumor responses is demonstrated in mouse tumor models where use of a CD226-neutralizing monoclonal antibody (mAb) abrogates the efficacy of combining mAbs against PD-L1 and TIGIT (Johnston et al., 2014). Furthermore, CD8^+^ T cells with decreased or loss of CD226 expression exhibit characteristics of dysfunction, and such cells are present in tumors and correlate with resistance to cancer immunotherapy (Braun et al., 2020; Weulersse et al., 2020). Thus, simultaneous blockade with anti-PD-(L)1 and anti-TIGIT mAbs may coordinately inhibit the negative signals of PD-1 and TIGIT by facilitating the activities of their respective client costimulatory receptors. It nevertheless remains unclear why targeting PD-1 and TIGIT pathways appear to synergize, especially since PD-(L)1 mAb, but not TIGIT mAb, can often yield complete responses on its own.

The CD28:CTLA-4 and CD226:TIGIT receptor pairs are highly analogous. CTLA-4 expression increases immediately following TCR engagement and attenuates CD28 signals by outcompeting CD28 for binding to, or depleting, their shared ligands (CD80, CD86) (Qin et al., 2019; Sansom and Walker, 2006; Wei et al., 2018). TIGIT expression increases following T cell activation and attenuates activating signals through CD226 by outcompeting CD226 for binding to their shared ligands PVR (CD155) and PVRL2 (CD112) (Manieri et al., 2017). Recent evidence, however, suggests that inhibition of costimulatory molecules is more sophisticated than originally thought. Both CD28 and CD226 are not only regulated by their respective inhibitory receptors (i.e., CTLA-4 and TIGIT) but may also be dephosphorylated by the Shp2 phosphatase that is recruited to the PD-1 intracellular domain (ICD) following T cell activation and phosphorylation by Lck (Hui et al., 2017; Wang et al., 2018).

TIGIT and PD-1 are often considered as markers of exhausted CD8^+^ T cells, enabling the maintenance of a quiescent state in these cells. However, expression of these inhibitory receptors is increased upon T cell activation and exhibits complex patterns among various T cell subsets (Chauvin and Zarour, 2020; Hashimoto et al., 2018; Wu et al., 2020; Zarour, 2016). Rather than maintaining exhaustion, there is increasing evidence that PD-1 acts to restrain the expansion of antigen-specific T cells at the priming or expansion step (Im et al., 2016; Miller et al., 2019; Oh et al., 2020; Siddiqui et al., 2019; Utzschneider et al., 2016; Wu et al., 2016; Yost et al., 2021). TIGIT and PD-1 may also be co-expressed by CD103^+^ CD8^+^ resident memory T cells (Trm), a T cell subpopulation that mediates anti-tumor immunity that may be derived from stem cell-like memory T (Tscm) or effector T (Teff) cells (Corgnac et al., 2020; Craig et al., 2020; Park et al., 2019).

In this study, we investigated how PD-(L)1 or TIGIT blockade regulated CD8^+^ T cell responses against tumors. CD226 expression was required for efficacy of PD-(L)1 or TIGIT targeted therapies and PD-1 and TIGIT converged on CD226 to disable activation of this key costimulatory receptor. Thus, these two inhibitory receptors are interdependent, suggesting that their coordinate inhibition is required to elicit optimal T cell activity.

## RESULTS

### Absence of CD226 diminishes anti-tumor efficacy with PD-1 or TIGIT checkpoint blockade

**Figure 1.**
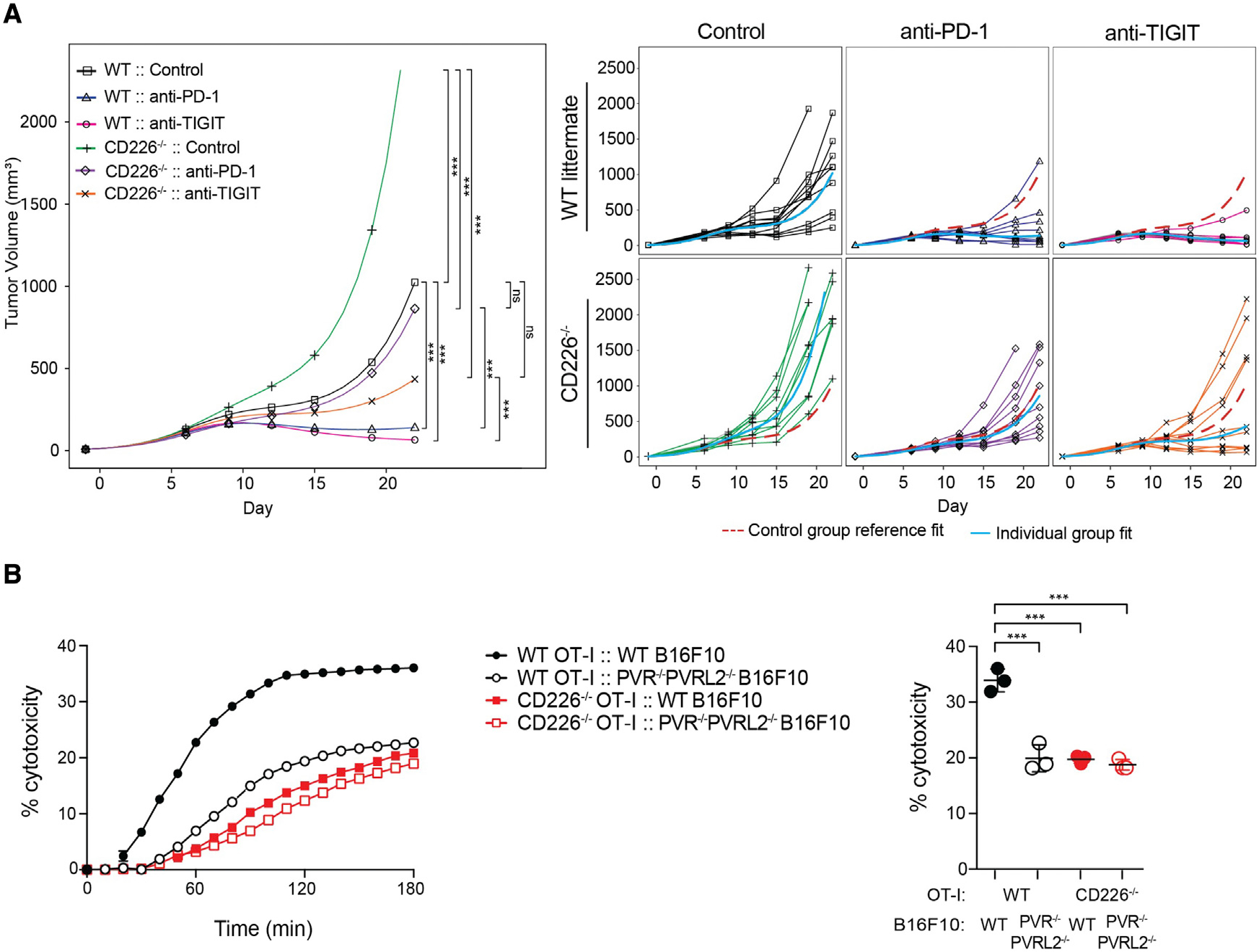
CD226 deficiency reduces efficacy of PD-1 and TIGIT checkpoint blockade and impairs CD8^+^ T cell responses (A) BALB/c WT or *Cd226*^−/−^ mice inoculated with syngeneic CT26 tumor cells and treated with isotype control, anti-PD-1, or anti-TIGIT antibodies. Tumor growth was monitored and grouped analysis and growth curves for each individual animal (n = 10 per group) are shown. Tumor volume remaining below 32 mm^3^ was considered to be a complete response (CR). p values are shown for end of study at day 23 using two-way ANOVA test with post hoc Tukey’s multiple comparisons; *p < 0.05; **p < 0.01; ***p < 0.001; ns, not significant. (B) CD226 deficiency impairs cytotoxicity of CD8^+^ T cells. WT OT-I cells (black lines) or *Cd226*^−/−^ OT-I cells (red lines) were used as effector cells against B16F10 melanoma or PVR/PVRL2-deficient (PVR^−/−^PVRL2^−/−^) B16F10 target cells pulsed with OVA (SIINFEKL) peptide. Representative real-time profiling of killing is shown on the left panel. Scatterplot (right panel) shows percent cytotoxicity at the 3 h time point. Data are shown as mean ± SD of three independent experiments. p values are shown for one-way ANOVA test with post hoc Tukey’s multiple comparisons; *p < 0.05; **p < 0.01; ***p < 0.0001.

We previously observed that the anti-tumor efficacy of combined anti-PD-L1 and anti-TIGIT blockade in mice is abrogated by a CD226-blocking mAb (Johnston et al., 2014), suggesting that costimulation by both CD226 and CD28 is required to overcome inhibition by PD-1 and TIGIT. To understand this relationship in greater detail, we monitored the growth of syngeneic CT26 tumor cells in wild-type (WT) or *Cd226*^−/−^ mice. To focus the analysis selectively on TIGIT or PD-1, we examined tumor growth in the prevention setting (i.e., the mAb was administered shortly after tumor implantation) where both anti-TIGIT and anti-PD-L1 exhibit single agent activity; in the therapeutic setting, anti-TIGIT is most effective when combined with anti-PD-L1. As shown in Figure 1A and in agreement with recent results (Weulersse et al., 2020), WT mice that received anti-PD-1 or anti-TIGIT exhibited reduced tumor growth compared to animals treated with a control antibody. However, the therapeutic benefit of either anti-PD-1 or anti-TIGIT was reduced or lost in *Cd226*^−/−^ mice even though signaling via CD28 remained intact.

As CD226 deficiency is known to alter the transcriptional profile of CD8^+^ T cells affecting the effector program (Braun et al., 2020; Du et al., 2018; Weulersse et al., 2020), we also assessed the requirement for CD226 to mediate target cell killing. When OT-I CD8^+^ T cells from WT or *Cd226*^−/−^ mice were co-cultured with OVA (SIINFEKL) peptide loaded B16F10 melanoma cells expressing or deficient in both PVR and PVRL2, *Cd226*^−/−^ CD8^+^ T cells exhibited a reduced capacity to kill antigen-loaded melanoma cells in a PVR- and/or PVRL2-dependent manner as compared to WT CD8^+^ T cells (Figure 1B). Taken together, these results confirm a role for PVR and/or PVRL2 regulation of CD226 signaling in mediating CD8^+^ T cell responses, a role that is controlled not only by TIGIT but also by PD-1.

### CD226 expression is associated with clinical response to anti-PD-L1 immunotherapy

We next asked whether CD226 expression was also a determinant for clinical responses to atezolizumab, an anti-PD-L1 mAb, in patients with non-small cell lung carcinoma (NSCLC) (Fehrenbacher et al., 2016; Peters et al., 2017; Rittmeyer et al., 2017). We analyzed bulk RNA-seq from NSCLC tumor samples from three randomized clinical trials with atezolizumab and assessed how gene expression of *CD226*, *PDCD1* (encoding PD-1), and *TIGIT* associated with clinical outcomes. We included the expression of *CD28* in the analysis, as CD28 is regulated by the PD-1/PD-L1 pathway (Hui et al., 2017; Kamphorst et al., 2017).

**Figure 2.**
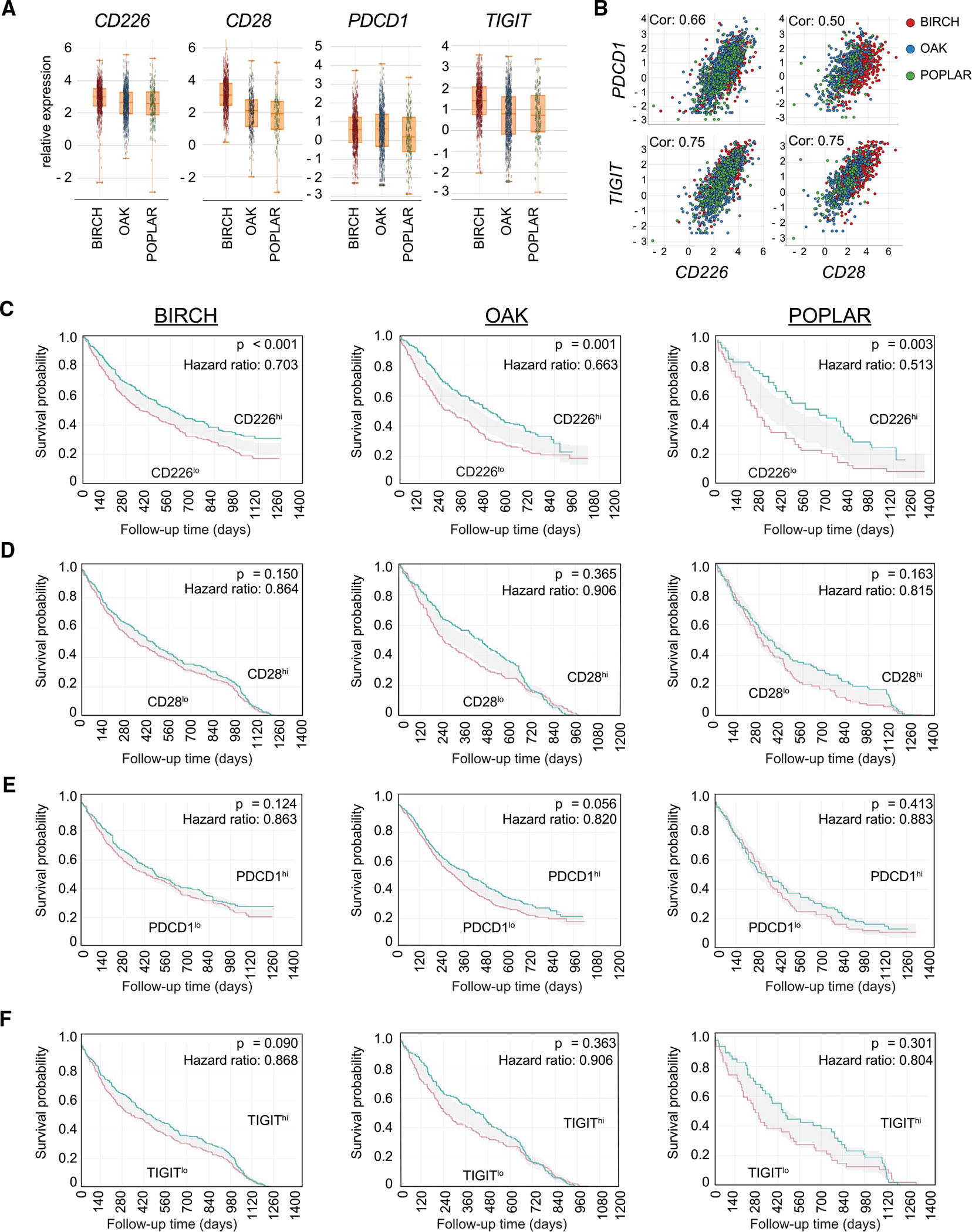
CD226 expression and association with clinical response to PD-L1 checkpoint blockade treatment in NSCLC (A) Relative gene expression of *CD226, CD28, PDCD1,* or *TIGIT* in three different atezolizumab clinical trials (BIRCH, OAK, or POPLAR) in NSCLC. (B) Co-expression of *CD226* (left) or *CD28* (right) with *PDCD1* or *TIGIT* are positively correlated. Shown is the Spearman correlation coefficient. (C–F) Survival based on *CD226* (C), *CD28* (D), *PDCD1* (E), or *TIGIT* (F) gene expression. Kaplan-Meier plots of overall survival (OS) are shown for the atezolizumab arm in the indicated clinical trial (BIRCH, OAK, or POPLAR), with patients separated on the basis of gene expression. Patients were dichotimized into top 50% (high, green line) or bottom 50% (low, red line) relative to the median expression over all patients in the corresponding clinical trial. Two-sided p values and hazard ratios from a Cox proportional hazards model are indicated for each plot.

Expression of *CD226, CD28, PDCD1,* and *TIGIT* in each trial exhibited a wide range (Figure 2A), but the expression of *CD226* and *CD28* was closely correlated with *PDCD1* (correlation: 0.66 and 0.50, respectively) and *TIGIT* (correlation: 0.75 and 0.75, respectively) (Figure 2B). Stratification of patients in each clinical trial based on intratumoral *CD226* expression indicated an association between expression and overall survival (OS) (BIRCH: HR 0.703, p < 0.001; OAK: HR 0.663, p = 0.001; POPLAR: HR 0.513, p = 0.003) and improved progression-free survival (PFS) (BIRCH: HR 0.812, p = 0.013; OAK: HR 0.582, p < 0.001; POPLAR: HR 0.505, p = 0.001) (Figures 2C and S1A). Moreover, the positive correlation of *CD226* expression with PFS was not observed in the chemotherapeutic arms in any of these trials (Figure S1B). Elevated intratumoral *CD28* expression did not show an association with improved clinical benefit in any of these trials (Figure 2D), nor did the expression levels of other immune costimulatory molecules such as *ICOS*, *TNFRSF4* (OX-40), *TNFRSF9* (4–1BB), and *TNFRSF18* (GITR) (Figures S1C–S1F). *PDCD1* or *TIGIT* expression also failed to associate with clinical response (Figures 2E and 2F). Together, these data indicate that CD226 expression, but not CD28 expression, is a correlate of anti-PD-L1 response in cancer.

### NSCLC tumor-infiltrating CD8^+^ T cells co-expressing CD226, PD-1, and TIGIT mark a Trm population

**Figure 3.**
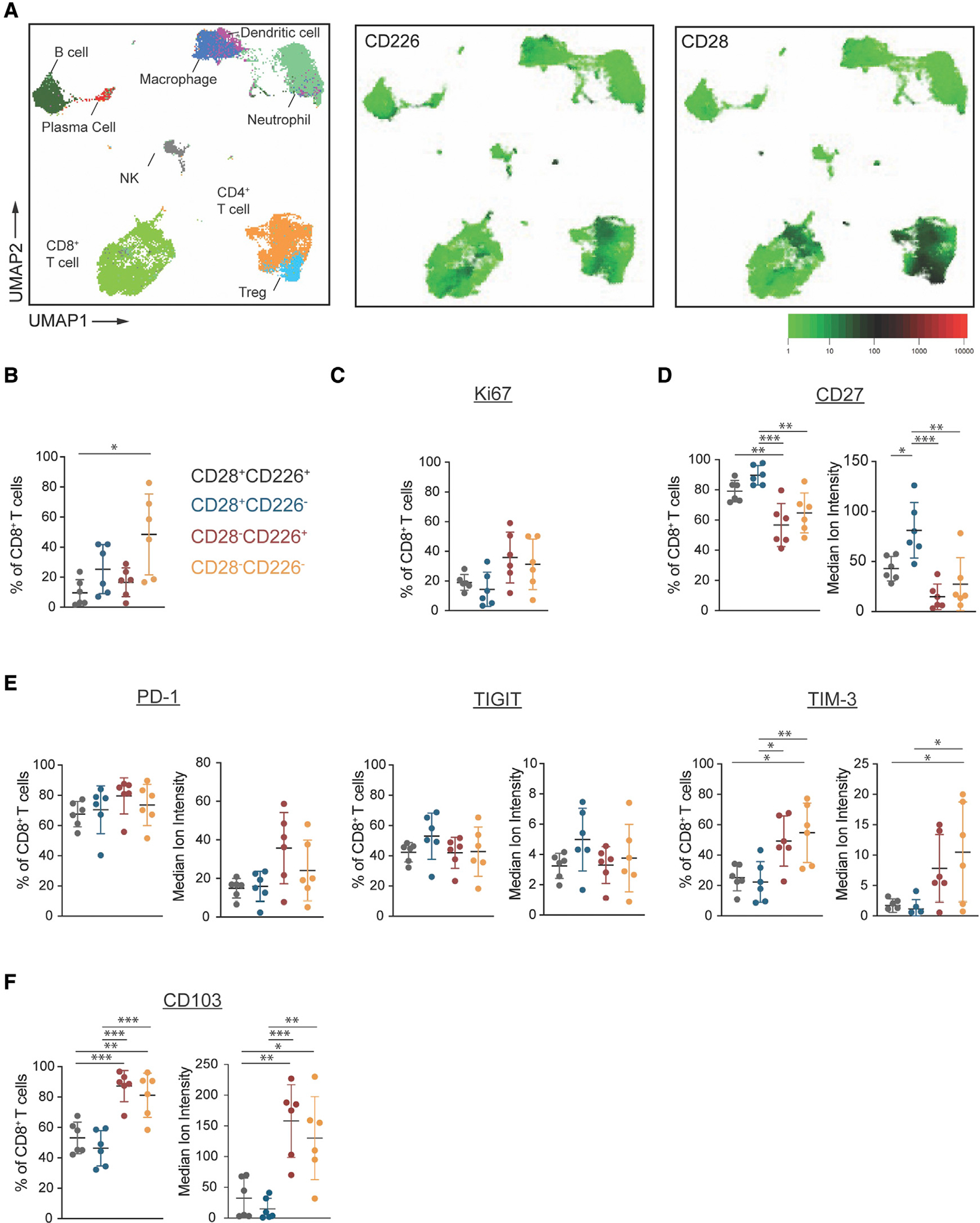
Expression of CD226 and CD28 in CD8^+^ T cells by CyTOF in NSCLC tumors (A) CD45^+^ cells from NSCLC patient tumors (n = 6) were analyzed by CyTOF. 8,000 downloaded cells per sample were aggregated and clusters generated in an unsupervised manner by uniform approximation and projection (UMAP). Immune cell populations were defined by manual gating and projected onto the UMAP (left). Expression of CD226 (middle) or CD28 (right) across total CD45^+^ cells was overlaid onto the UMAP. (B) Frequencies of CD8^+^ T cell expressing CD226 and/or CD28 in NSCLC tumors. CD8^+^ T cells were subtyped by CD28^+^CD226^+^ (gray), CD28^+^CD226^−^ (blue), CD28^−^CD226^+^ (red), or CD28^−^CD226^−^ (yellow). (C–F) Comparison of expression by frequency (left), and where shown, median ion intensity (right) for indicated markers within CD8^+^ T cells gated on CD226 and/or CD28 expression. (C) Proliferation marker Ki67. (D) CD27. (E) Checkpoint inhibitors PD-1, TIGIT, and TIM-3. (F) Trm cell marker CD103. Whiskers denote mean ± SD (n = 6). p values are shown for one-way ANOVA with post hoc Tukey’s for multiple comparisons; *p < 0.05, **p < 0.01, ***p < 0.001.

We then profiled the distribution of CD226, CD28, PD-1, and TIGIT expression within NSCLC patient samples (Table S1) by high-dimensional time-of-flight mass cytometry (CyTOF). A 38-parameter panel was developed and used to identify immune cell lineage, markers of tissue residency, activation and inhibitory markers, and transcription and cytotoxic factors (Table S2). A uniform manifold approximation and projection (UMAP) was generated to resolve distinct immune cell populations (Figure 3A, left). CD226 and CD28 were expressed predominantly by CD4^+^ and CD8^+^ T cell subpopulations; however, the subpopulations only partially overlapped (Figure 3A, center and right).

To better characterize the subpopulations that expressed CD28 and CD226 either alone or together, CD8^+^ tumor-infiltrating lymphocytes (TILs) were gated to assess the phenotypic markers associated with all possible combinations of CD226 and CD28 expression. As shown in Figure 3B, each of the possible subtypes was detected, with the CD226^+^CD28^+^ double-positive cells being among the least frequent. All exhibited some level of proliferative potential, based on Ki67 expression (Figure 3C), but the CD226^+^CD28^+^ cells expressed high levels of CD27, a marker of antigen-specific expansion and early development of memory T cells (Figure 3D). They also exhibited higher levels and frequencies of PD-1 and TIGIT expression, as compared to the T cell exhaustion marker TIM-3 (Figure 3E). Although there was significant overlap, cell populations that were positive for CD28 (with or without coordinate expression of CD226) also expressed lower levels of CD103, a marker defining Trm cells (Figure 3F).

To obtain a more detailed phenotypic analysis of expression in CD8^+^ T cells, single-cell RNA sequencing (scRNA-seq) was performed on CD45^+^ cells isolated from the same NSCLC tumor samples used in the CyTOF analysis. CD8^+^ T cells from normal adjacent tissues (NAT) were also included for comparison.

**Figure 4.**
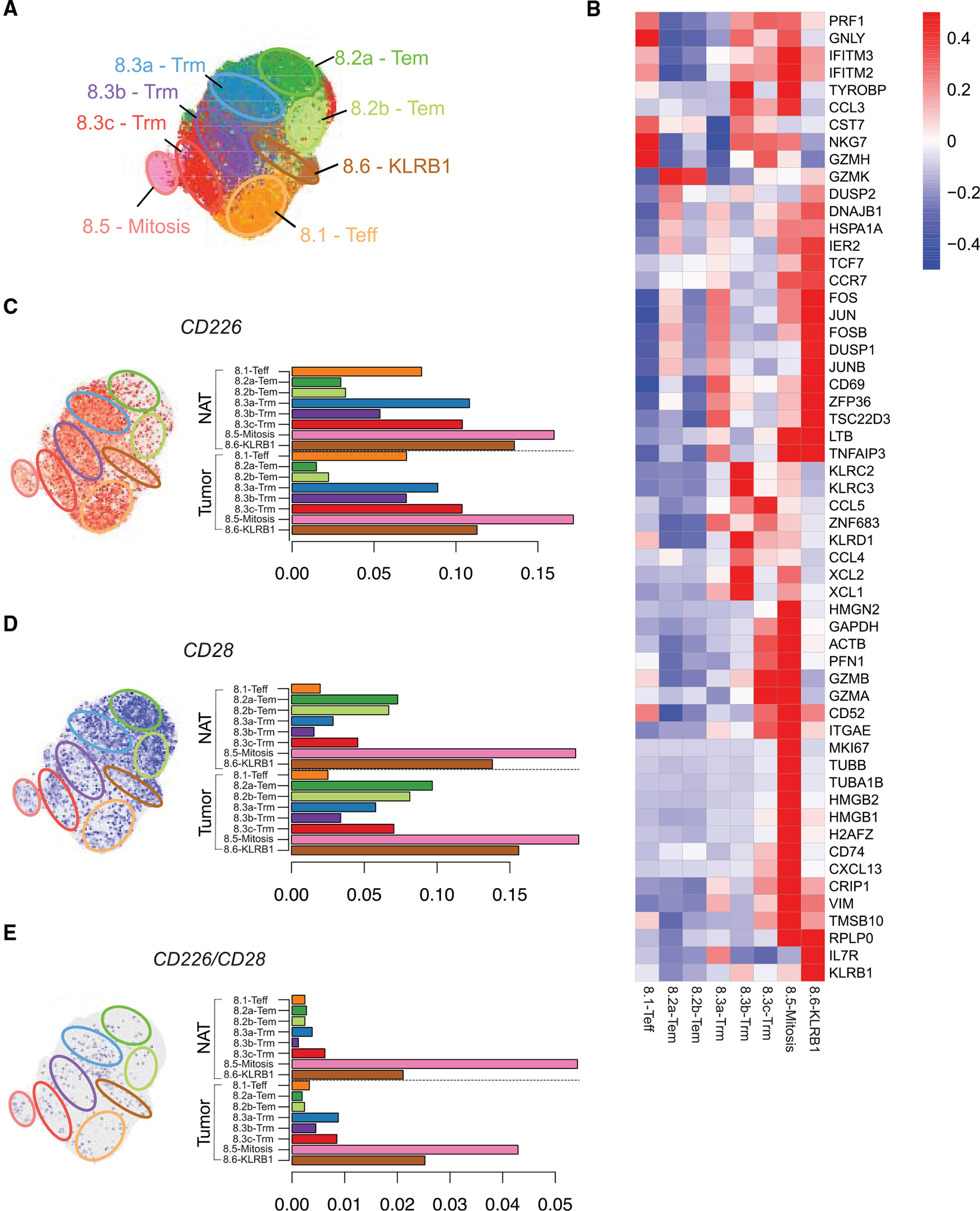
CD226 and CD28 expression in CD8^+^ T cells in NSCLC tumors by scRNA-seq (A) Cluster analysis was performed on scRNA-seq data of CD8^+^ T cells obtained from all six NSCLC patients and plotted by UMAP dimensionality reduction. Cluster assignments were generated by unsupervised clustering and colored by cluster assignment. Approximate regions of the clusters are demarcated with labeled ovals (top left). (B) Relative expression of T cell-associated genes across CD8^+^ clusters. (C–E) Individual CD8^+^ T cells are plotted within each UMAP (left) or plotted by bar graph to depict frequency (right) of CD226 (C), CD28 (D), or CD226 and CD28 (E) expression within each CD8^+^ T cell cluster.

Analysis of gene expression partitioned the CD8^+^ TILs into eight clusters by unsupervised clustering (Figure 4A) and hierarchical clustering using raw, unscaled counts data was used to visualize differential gene expression between clusters (Figure 4B). Annotations for each were based on published gene signatures (Banchereau et al., 2021; Wu et al., 2020): effector T cells (Teff; cluster 8.1) characterized by expression of cytotoxic proteins; effector memory T cell clusters (Tem; cluster 8.2a–b) defined by expression of high *GMZK* expression and bifurcated to represent activated Tem cells (8.2a) based on increased *JUN* and *FOS* expression or non-activated Tem cells (8.2b); three distinct resident memory T cell clusters (Trm; cluster 8.3a–c), defined by increased *ITGAE* and immune checkpoint expression; mitotic cells in a highly proliferative state, as evidenced by high *MKI67* expression, representing all clusters (mitosis; cluster 8.5); and cells that expressed *KLRB1* (KLRB1; cluster 8.6), a cluster that potentially encompasses a variety of cytotoxic memory cell types. KLRB1^+^ cells are described as being related to mucosal associated invariant T (MAIT) cells, Tc17 cells capable of producing IL-17, and cells with Tem or Temra (terminally differentiated, CD45RA^+^ Tem) properties. Notably, non-MAIT KLRB1^+^ cells also have features of Tscm cells (Han et al., 2020; Konduri et al., 2021). Tscm cell signature genes *TCF7*, *CCR7*, and *IL7R* were expressed at high levels within the KLRB1 cluster 8.6.

We then profiled the distribution of CD28 or CD226 among each of the CD8^+^ T cell clusters. In tumors and NAT, each costimulatory receptor was broadly expressed by all clusters, albeit to variable extents (Figures 4C and 4D). The similarities between tumor and NAT were consistent with our recent observation that clonotypic expansion of CD8^+^ T cells in tumors is also reflected in NAT (Wu et al., 2020). In general, the highest expression was found in cells in the mitotic (cluster 8.5) and KLRB1^+^ (cluster 8.6) clusters. Notable patterns of expression were observed in clusters 8.1 (Teff), 8.2a–b (Tem), and 8.3a–c (Trm). CD226 expression was higher than CD28 in Teff cells (odds ratio of CD226 expression relative to CD28 expression = 2.92; p < 0.0005) and Trm cells (odds ratio ranging from 1.53 to 2.14 in clusters 8.3a–c; p < 0.0005 in all Trm clusters), whereas CD28 expression was higher than CD226 in Tem cells (odds ratio = 0.14 for cluster 8.2a, 0.26 for cluster 8.2b; p < 0.0005 for both Tem clusters).

A different picture emerged when scoring clusters that were double positive for CD28 and CD226 (Figure 4E). Although individual cells in all clusters expressed one or the other marker, co-expression was rare in Tem or Trm cells. Double-positive CD8^+^ T cells were most prevalent in the 8.5 (mitosis) and 8.6 (KLRB1) clusters (Figure 4E), suggesting that cells poised for proliferation or activation were the ones expressing both CD28 and CD226.

Additionally, we profiled CD8^+^ T cells on the basis of CD226 and CD28 co-expression with PD-1 and TIGIT (Figure S2). Cells that were positive for all four markers were most frequently found in the mitotic cells (cluster 8.5).

### PD-1 and TIGIT converge to inhibit CD226 phosphorylation

Given the pattern of coordinate expression and the apparent functional synergy between both PD-1 and TIGIT inhibition with CD226 and CD28, we next asked whether there was an underlying biochemical relationship. We established a cell culture system using Jurkat cells transduced with CD226 alone or together with TIGIT and/or PD-1 (Figure S3). Raji cells expressing PD-L1, PVR, or both ligands were loaded with superantigen (SEE) and used as antigen-presenting cells (APCs) to activate Jurkat cells (Figure S3A). By immunoblot, we found that residue Y322, rather than S329, within the ICD of CD226 was the major phosphorylation site for CD226 in activated T cells (Figure S4A), consistent with a recent publication (Jin et al., 2020).

**Figure 5.**
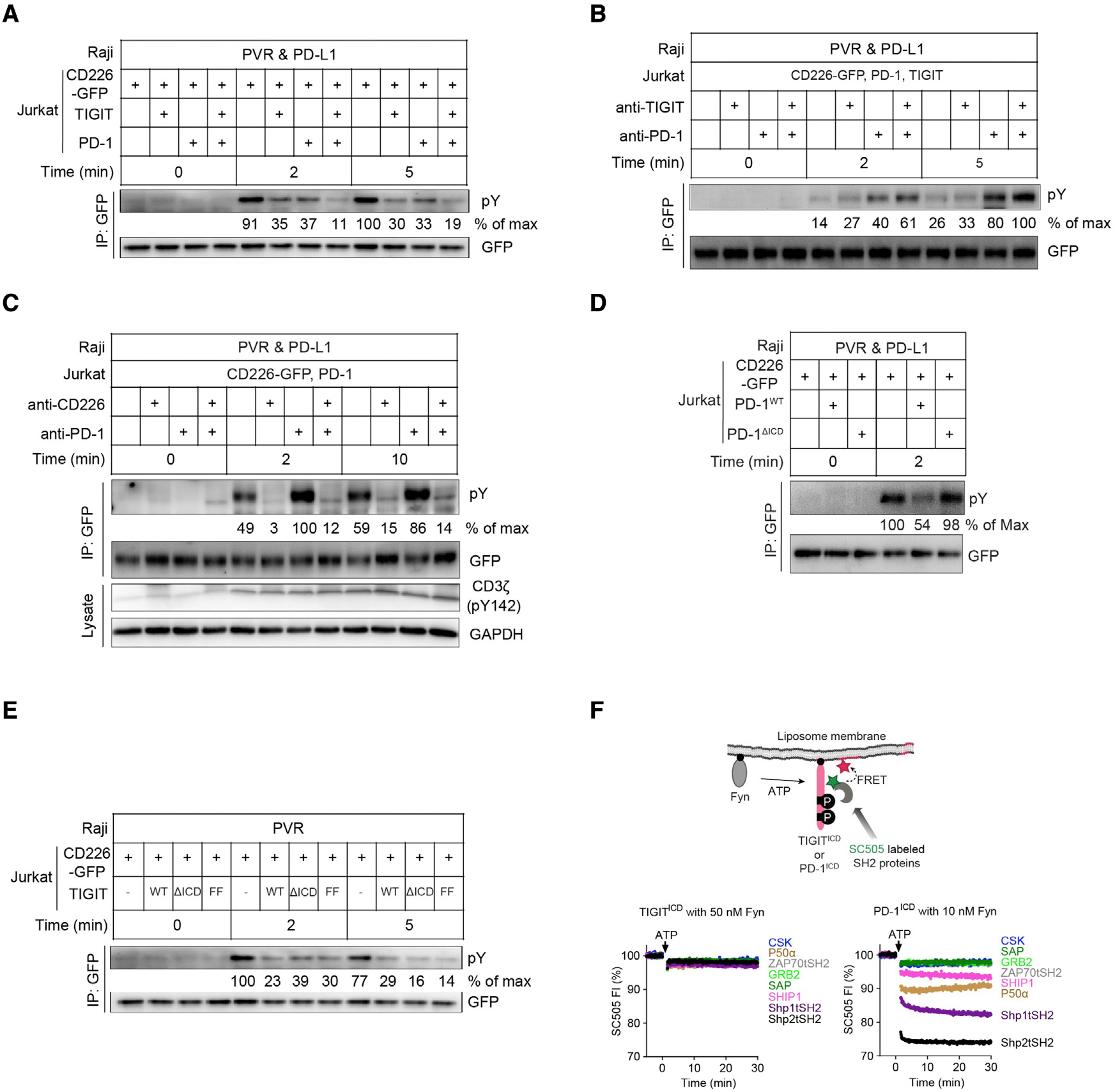
PD-1 and TIGIT converge to regulate CD226 through distinct mechanisms. (A–E) Representative phospho-tyrosine immunoblots showing CD226 phosphorylation upon co-culture of Jurkat cells expressing indicated receptors with SEE-loaded Raji cells expressing indicated ligands. In (B) and (C), Jurkat cells were pretreated with anti-TIGIT (10A7), anti-PD-1 (pembrolizumab), anti-CD226 (DX11), or in combination. CD226-GFP was enriched by GFP IP after lysing the Raji:Jurkat conjugates at the indicated time points. The “% of max” values under each blot denote the optical densities (OD) of the corresponding bands above normalized to the OD of the strongest band of the same blot. (F) Cartoon on top depicts a liposome-based FRET assay for measuring the recruitment of SC505-labeled SH2 proteins to liposome-reconstituted, Fyn-phosphorylated TIGIT^ICD^ or PD-1^ICD^. Shown on the bottom are representative time courses of SC505 FI before and after addition of 1 mM ATP for TIGIT^ICD^ (left) or PD-1^ICD^ (right). Green stars denote SC505 dye, red stars denote rhodamine-PE lipid, black circles denote His_10_ tags.

CD226^+^ Jurkat cells devoid of PD-1 and TIGIT expression exhibited robust phosphorylation of CD226 (pCD226) within 2 min after stimulation by PD-L1^+^PVR^+^ SEE-loaded Raji cells. Either PD-1 or TIGIT expression in the Jurkat cells decreased pCD226, suggesting that PD-1 and TIGIT could independently inhibit CD226. When both PD-1 and TIGIT were expressed, however, far less pCD226 was detected, demonstrating that PD-1 and TIGIT acted together to regulate CD226 activation (Figure 5A). Adding either anti-PD-1 or anti-TIGIT to the cells partially restored pCD226, although anti-PD-1 appeared to do so more efficiently. Providing anti-PD-1 and anti-TIGIT simultaneously further enhanced the appearance of pCD226 (Figure 5B).

### The ICD of PD-1, but not that of TIGIT, is required for inhibition of CD226 phosphorylation

We next examined the ability of PD-1 to regulate CD226 phosphorylation in the absence of TIGIT. A previous study showed that liposome-reconstituted PD-1:Shp2 complex can dephosphorylate the CD226 ICD (Wang et al., 2018); however, the extent to which PD-1 regulates CD226 phosphorylation in intact T cells is unknown. As shown in Figure 5C, CD226^+^PD-1^+^ Jurkat cells exhibited some level of pCD226 after exposure to PVR^+^PD-L1^+^ SEE-loaded Raji cells. In response to PD-1 blockade, the level of pCD226 increased 1.5- to 2.0-fold at both the 2- and 10-min time points. This effect was dependent on PVR binding to CD226, as a CD226-blocking mAb abolished the effect. We observed no systematic differences in CD3ζ phosphorylation under any of the conditions tested. These data demonstrated that the PD-L1 and PD-1 signaling pathway inhibited PVR-induced CD226 phosphorylation. This effect was also found to require the ICD of PD-1 (PD-1^ICD^), as expression of a PD-1 mutant lacking an ICD (PD-1^ΔICD^) failed to decrease the amount of pCD226 after stimulation (Figure 5D). The ICD requirement suggested that PD-1 inhibits CD226 phosphorylation via its intracellular effectors, e.g., Shp2 phosphatase, which was recruited by the PD-1^ICD^ (Hui et al., 2017; Wang et al., 2018).

We next investigated how TIGIT regulates CD226 signaling. Similar to PD-1, the ICD of TIGIT (TIGIT^ICD^) contains two tyrosines embedded in an immunoglobulin tail tyrosine (ITT) motif and an immunoreceptor tyrosine inhibitory motif (ITIM). Given the abilities of several ITIM-containing receptors to recruit SH2-containing phosphatases such as Shp1 and Shp2, it is tempting to presume that TIGIT recruits Shp1 or Shp2 in an analogous fashion. However, unlike PD-1^ICD^, the TIGIT^ICD^ lacks an associated immunoreceptor tyrosine-based switch motif (ITSM), which could limit effector recruitment. To determine the role of TIGIT^ICD^ in regulating CD226 phosphorylation, we co-expressed CD226 with WT TIGIT (TIGIT^WT^), a TIGIT mutant devoid of an ICD (TIGIT^ΔICD^), or a full-length TIGIT mutant with both phosphorylatable tyrosines altered to phenylalanines (TIGIT^FF^) in Jurkat cells. Upon the addition of PVR^+^ SEE-loaded Raji cells, pCD226 was inhibited by both WT TIGIT and TIGIT mutants to similar extents (Figure 5E). Thus, in contrast to PD-1, the TIGIT^ICD^ was dispensable for its inhibitory effect on pCD226.

As the TIGIT^ICD^ was not required to inhibit CD226 phosphorylation, we turned to our previously described cell-free liposome reconstitution system to determine whether the TIGIT^ICD^ had any ability to recruit important effector molecules using Föster Resonance Energy Transfer (FRET) as a readout, where any recruitment of purified, recombinant effectors from the extravesicular solution to the receptor-anchored liposomes would result in quenching of the donor fluorescence of the effectors by liposome-embedded acceptor fluorophores (Hui and Vale, 2014) (see material and methods). We first conducted a careful titration of the kinase Fyn to determine the Fyn concentration required for efficient TIGIT^ICD^ and PD-1^ICD^ phosphorylation at the time course of this assay. Anti-phosphotyrosine (pY) IB revealed that when PD^ICD^ or TIGIT^ICD^ was reconstituted to liposomes together with Fyn, both ICDs could be phosphorylated by Fyn in a dose-dependent manner, though PD-1 was a better substrate (Figure S4B). Based on these results, we elected to use 10 nM and 50 nM Fyn to phosphorylate PD-1^ICD^ and TIGIT^ICD^, respectively, in the effector recruitment assay. As shown in Figure 5F, in contrast to the PD-1^ICD^, TIGIT^ICD^ failed to recruit any of the common T cell signaling proteins tested, including Shp1, Shp2, ZAP70, Grb2, SHIP-1, or P50α (the regulatory subunit of PI3K). Under the same condition, the PD^ICD^ recruited Shp2, and to a lesser extent, Shp1 and P50α.

In a complementary and unbiased but less quantitative assay, we pre-phosphorylated GST-tagged TIGIT^ICD^ and PD-1^ICD^ and used either of these as a bait to pull down proteins from the lysate of Raji:Jurkat conjugates and identified the proteins using mass spectrometry (MS), as described (Xu et al., 2020). As expected, MS detected phosphorylation of both tyrosines within the TIGIT^ICD^: Y225 in ITT and Y231 in ITIM, but phosphorylation of Y231 within the ITIM appeared to be much weaker (Figure S4C), consistent with a previous study in HEK293A cells (Liu et al., 2013). Similar to the liposome reconstitution assay, PD-1^ICD^ enriched SH2-containing proteins including Shp1 and Shp2 as compared to the tyrosine-mutated negative control (PD-1^ICD-FF^), whereas TIGIT^ICD^ failed to pull down either Shp1 or Shp2 (Figure S4D).

Taken together, these data suggest that PD-1 inhibited CD226 phosphorylation by recruiting Shp2 to its ICD, while the TIGIT^ICD^ is dispensable for its inhibitory effect on pCD226, perhaps due to the inefficient phosphorylation of the TIGIT ITIM.

### The TIGIT extracellular domain inhibits PVR-CD226 interaction and CD226 homodimerization

**Figure 6.**
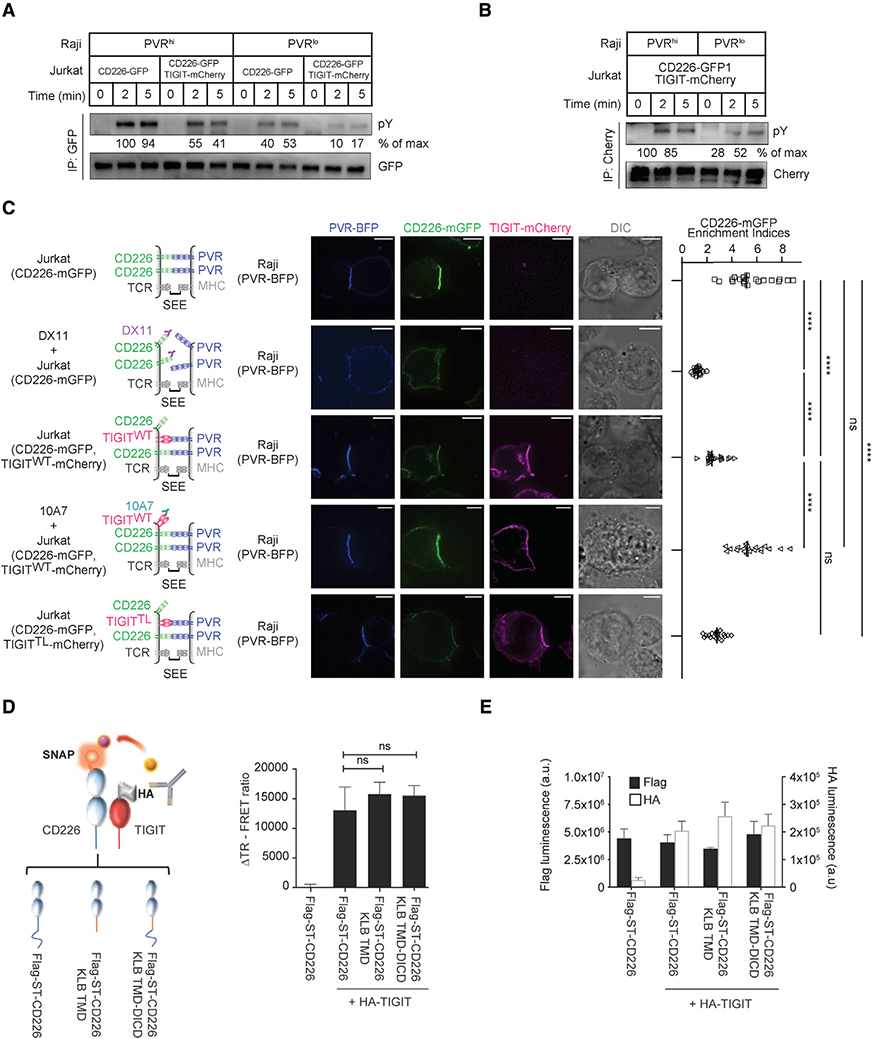
TIGIT inhibits CD226 through lateral associations through ECDs (A and B) Representative immunoblots showing the degrees of CD226 and TIGIT phosphorylation (pY) after co-culturing Jurkat cells expressing indicated receptors and SEE-loaded Raji cells expressing indicated ligands. Times denote the duration of co-culture before lysis. CD226-GFP and TIGIT-mCherry were captured using GFP IP and mCherry IP, respectively. The “% of max” values under each blot denote the OD of the corresponding bands above normalized to the OD of the strongest band of the same blot. (C) Confocal images of Jurkat:Raji cell conjugates probing the effects of TIGIT on PVR-mediated CD226 accumulation to the Raji:Jurkat interface. Left: depiction of the relevant proteins at the cell conjugate. Middle: confocal and differential interference contrast (DIC) images of a Raji:Jurkat conjugate acquired five minutes after contact. Right: scatterplot summarizing CD226 enrichment indices of the five conditions (means ± SD, n = 20 conjugates from three independent experiments). Scale bars, 5 μm. ****p < 0.0001; ns, not significant; Student’s t test (n = 20). (D and E) Interaction studies between CD226 and TIGIT using FRET. (D) Cell surface TR-FRET signal between acceptor labeled full-length or chimeric Flag-ST-CD226 and donor labeled full length HA-TIGIT. (E) Cell surface expression of Flag-ST-CD226 constructs (black bar) and HA-TIGIT (white bar) as measured by ELISA. Data are average of 2 independent experiments, each performed in triplicate, and shown as mean ± SEM ns = not significant.

We next focused on the extracellular domain (ECD) of TIGIT, which is known to compete with CD226 for binding to their shared ligand PVR (Johnston et al., 2014; Stengel et al., 2012; Yu et al., 2009). To determine whether ligand competition alone was responsible for TIGIT-mediated inhibition of CD226 signaling, we used Raji cells expressing either low or high amounts of PVR (Figure S3B). We reasoned that the ligand competition effect would be more evident when ligand was limiting. The degree of CD226 phosphorylation in Jurkat cells was greater at high as opposed to low PVR expression on Raji cells, at both 2- and 5-min time points (Figure 6A; PVR^hi^ versus PVR^lo^). Although co-expression of TIGIT with CD226 decreased pCD226 signal under both PVR^hi^ and PVR^lo^ conditions, the decrease in pCD226 was more pronounced under PVR^lo^ conditions, consistent with the ligand-competition model. Also consistent with the model was the observation that TIGIT phosphorylation was induced by PVR in a dose-dependent manner (Figure 6B).

To characterize PVR:CD226 and PVR:TIGIT interactions at the Raji:Jurkat interface, we performed confocal microscopy on Raji:Jurkat couplets to determine whether PVR-induced CD226 and TIGIT exhibited polarization at the site of cell-cell contact. In the absence of PVR^+^ Raji cells, CD226 was observed along the entire perimeter of CD226^+^ Jurkat cells, regardless of whether TIGIT was co-expressed (Figure S5). When CD226^+^ Jurkat cells encountered PVR^+^ Raji cells, CD226 was enriched to the Raji:Jurkat interface (Figure 6C, top row). This enrichment was ablated by DX11, an anti-CD226 mAb that blocks PVR:CD226 interaction (Figure 6C, second row). Thus, the polarized enrichment of CD226 was driven by trans-PVR:CD226 interaction. TIGIT co-expression significantly decreased CD226 polarization (Figure 6C, third row), consistent with the possibility that TIGIT disrupts or weakens CD226:PVR interaction. TIGIT was enriched to the Raji:Jurkat interface (Figure 6C, third row, mCherry channel), indicating that TIGIT sequestered PVR away from CD226. Blockade of TIGIT:PVR via anti-TIGIT mAb (10A7) restored the CD226 enrichment at the cell-cell contact site (Figure 6C, fourth row). Finally, expression of the TIGIT^ΔICD^ mutant decreased CD226 enrichment at the interface to a similar extent as the expression of TIGIT^WT^, providing further evidence that the TIGIT^ICD^ was not required for the regulation of CD226 (Figure 6C, bottom row).

These findings suggested that the reported abilities of TIGIT to disrupt CD226 homodimerization (Johnston et al., 2014) may also not involve ICD interactions. To test this possibility, we transfected cells with full-length or chimeric Flag-SNAP tagged (ST) CD226 with or without HA-TIGIT. We then monitored the interaction between the different pairs of receptors on the cell surface using time-resolved FRET (TR-FRET). Flag-ST-CD226 in combination with HA-TIGIT yielded a significant TR-FRET signal as compared to Flag-ST-CD226 alone (Figure 6D). Similar results were obtained using a CD226 construct where the transmembrane domain (TMD) and ICD were deleted and replaced either with a TMD from an irrelevant receptor or with full-length CD226 containing an irrelevant TMD fused to the CD226 cytoplasmic domain. Cell surface expression of the immunoreceptors was consistent across all the combinations tested as shown by ELISA (Figure 6E). These results suggested that TIGIT and CD226 interacted through their corresponding ECD and that neither the TMD nor ICD were essential for this interaction.

To gain further insight into the subcellular localizations of CD226, TIGIT, and PD-1, we employed total internal reflection fluorescence (TIRF) microscopy to visualize their microclusters at a hybrid synapse formed between a Jurkat cell expressing fluorescently tagged receptors of interest and a supported lipid bilayer (SLB) functionalized with anti-CD3ε, PVR, and PD-L1. These experiments revealed that all three receptors (CD226, TIGIT, and PD-1) colocalized as microclusters in the presence of their ligands (Figure S6A), consistent with their biochemical and functional crosstalk revealed in other experiments. Blockade of TIGIT:PVR and PD-1:PD-L1 interaction using anti-TIGIT or anti-PD-1 mAbs impaired TIGIT clustering and PD-1 clustering, respectively, and in both cases, decreased their degrees of colocalization with CD226 (Figures S6B and S6C).

Together, these data suggest that PD-1 and TIGIT pathways converge to inhibit CD226 co-stimulation. PD-1 repressed CD226 signaling directly through ICD-mediated dephosphorylation of CD226, while TIGIT inhibited CD226 through ECD-mediated impairment of CD226 dimerization and synaptic localization.

## DISCUSSION

Combination blockade of the PD-1 and TIGIT coinhibitory receptor pathways provides near complete elimination of large established tumors in a variety of preclinical mouse models (Johnston et al., 2014). The combination also reveals promising survival data in cancer patients, including in a randomized human clinical trial for NSCLC (Rodriguez-Abreu et al., 2020). In both mouse and humans, however, inhibition of TIGIT alone elicits far less impressive results. Why inhibiting both coinhibitory receptor pathways is superior to inhibiting them individually is a vexing question. We have identified a mechanistic explanation by showing that PD-1 and TIGIT pathways converge unexpectedly to regulate the critical costimulatory receptor CD226. At the cellular level, scRNA-seq and CyTOF of NSCLC patients revealed that while PD-1 and TIGIT were expressed throughout the CD8^+^ T cell compartment, CD226 and CD28 were co-expressed by distinct populations likely important for tumor immunity. At the molecular level, we demonstrated that PD-1 and TIGIT regulated CD226 activation through discrete but synergistic mechanisms not only *in vitro* but also in intact cells.

While TIGIT and PD-1 can independently regulate CD226, coordinate blockade of both inhibitory receptors was required to fully restore CD226 signaling. The ability of TIGIT to inhibit CD226 by competing for the shared ligand PVR was expected, but the TIGIT ICD had no role in controlling CD226 phosphorylation despite TIGIT possessing a putative ITIM motif. Instead, CD226 was dephosphorylated by Shp2 recruited to PD-1 akin to CD28 (Hui et al., 2017; Wang et al., 2018). The abilities of PD-1 and TIGIT to both inhibit CD226 might be required to efficiently turn off CD226 activation. Unlike CD28’s ligands (CD80/CD86), whose expression is limited to immune cells, PVR expression is more widely distributed. If PVR is expressed in excess of TIGIT, CD226 would become activated were it not for the ability of PD-1 to restrain CD226 phosphorylation via Shp2.

The functional importance of coordinate regulation was emphasized by the heterogeneity of CD226 and CD28 expression. While PD-1 and TIGIT were found in nearly all CD8^+^ T cell subpopulations, CD226 was expressed preferentially in Trm cells while CD28 was more prevalent in Teff cells and Tem cells. Trm cells are important mediators of anti-tumor immunity (Craig et al., 2020; Park et al., 2019) and may be derived from Tscm cells or lymph node-activated and recirculating Teff cells (Amsen et al., 2018; Corgnac et al., 2020). Yet, even though Trm cells can lack CD28 expression, activation of this T cell subpopulation may still require co-blockade of PD-1 and TIGIT due to PD-1’s ability to repress CD226 activity in the absence of TIGIT, and vice versa. The expression pattern of CD226 and CD28 in TILs may reflect a differentiation trajectory. Conceivably, the loss of CD28 on CD8^+^ TILs marks a more terminally differentiated state, with CD28^−^CD226^+^ TILs retaining the potential for cytotoxic function while CD28^−^CD226^−^ TILs have reached terminal exhaustion and may be unable to respond to anti-TIGIT plus anti-PD-L1 combination therapy. As CD28 and CD226 provide two important co-stimulatory pathways, loss of both may mark cells no longer capable of exerting anti-tumor effector function. In this context, it is important to again emphasize that PVR is widely expressed, even by most tumor cells, suggesting that CD226 activation may participate in the T cell effector activity more so than CD28, whose activating ligand is limited to immune cells.

Coordinate expression of CD28 and CD226 was also observed, but predominantly in only two clusters of TILs, namely the 8.5 (mitosis) and 8.6 (KLRB1) cluster subpopulations. Tscm cells, which appear to be contained in cluster 8.6 (KLRB1) on the basis of signature gene expression, have the capacity for self-renewal and to differentiate into memory and effector cells, thereby acting as a “resource” cell to replenish tumor-reactive CD8^+^ T cells (Brummelman et al., 2018; Gattinoni et al., 2011; Im et al., 2016; Miller et al., 2019; Siddiqui et al., 2019). The 8.5 (mitosis) cluster includes cells poised for proliferation. Defining the functional significance of co-expression in these two clusters will require additional work, although it is intriguing that treatment with anti-PD-1 results in a proliferative burst in CD226^+^ but not CD226^−^CD8^+^ T cells (Weulersse et al., 2020).

Recent evidence supports a mechanism whereby PD-1 blockade acts to induce T cell activation or proliferation during antigen presentation by dendritic cells in a fashion that also depends on CD28 stimulation by CD80 or CD86 (Kamphorst et al., 2017; Oh et al., 2020; Yost et al., 2019, 2021). Given the convergence between the PD-1 and TIGIT inhibitory pathways we have observed, it seems reasonable to speculate that CD226^+^CD28^+^ T cells will emerge as a relevant target population for checkpoint blockade. It remains possible that complete or partial reversal of an exhausted phenotype in CD8^+^ or CD4^+^ T cells may also contribute (Balança et al., 2021), but it is important to note that in mouse tumors, exhausted T cells lack CD226 expression and therefore might not be expected to respond functionally to PD-1 blockade (Braun et al., 2020; Weulersse et al., 2020). The anatomical site of T cell activation or exhaustion reversal by checkpoint blockade remains uncertain, although evidence in human and mouse suggests that it occurs in draining lymph nodes, tertiary lymphoid structures, or intratumoral lymphoid aggregates as opposed to tumor nests per se (Jansen et al., 2019; Siddiqui et al., 2019; Wu et al., 2020).

How the preferential usage of different costimulatory signals may elicit functionally specific or compensatory CD8^+^ T cell activities also remains to be understood. The recent identification of a family with a genetic defect in CD28 demonstrated the surprising finding that affected individuals were generally healthy but for a greatly enhanced sensitivity to human papillomavirus infection, suggesting compensatory functions between costimulatory receptors (Béziat et al., 2021). Further emphasizing the potential interaction among diverse costimulatory and inhibitory pathways are observations from preclinical studies demonstrating that efficacy of anti-CD137, anti-GITR, and anti-CTLA-4 required CD226 for anti-tumor activity (Wang et al., 2018; Weulersse et al., 2020).

Although there may be functional redundancy of signaling pathways, the combination of anti-PD-1 and anti-TIGIT blockade itself restored signals in a fashion that was mechanistically non-redundant. Recent biochemical studies have indicated that PD-1-Shp2 complexes target both CD28 and CD226 for dephosphorylation (Hui et al., 2017; Wang et al., 2018). However, these data relied solely on cell-free liposomal reconstitution and did not directly examine the role of TIGIT. We found that the ability of TIGIT to inhibit CD226 did not require its ITIM-bearing ICD, but blocked CD226 signaling by competing for ligand binding. The TIGIT ICD was phosphorylated in response to T cell activation, and MS analyses suggested that the ITT motif is a much better Src family kinase (SFK) substrate than the ITIM. Indeed, the Y+1 position of TIGIT ITIM is an arginine, which was enriched in low-efficiency SFK substrate sequence in a prior study (Shah et al., 2018). The poor phosphorylation of ITIM, in conjunction with the lack of ITSM, might explain the inability of TIGIT to recruit Shp1 or Shp2 and the undetectable contribution of the ICD to TIGIT suppression of CD226. Although TIGIT phosphorylation did not affect the regulation of CD226 activity in T cells, it might contribute to TIGIT activity in other cell types, such as T regulatory cells or NK cells, where its negative signal acts to maintain the inhibitory function of Treg cells or restrains NK effector function (Joller et al., 2011, 2014; Kurtulus et al., 2015; Li et al., 2014; Liu et al., 2013; Lozano et al., 2012; Lucca et al., 2019; Stanietsky et al., 2013; Zhang et al., 2018).

In summary, we have revealed an interplay between costimulatory and coinhibitory receptors, and places CD226 as a central player that may predict and dictate the successful outcome of immune checkpoint inhibition. CD226 is an activating receptor upon which multiple checkpoint inhibitor pathways converge, and thus is likely to be critical in generating optimal anti-tumor CD8^+^ T cell responses. Our findings help define the mechanistic rationale for combining TIGIT with PD-1 or PD-L1 blockade in the clinic, a combination that has thus far exhibited promising results in human cancer. While CD226 and CD28 are both clients of PD-1-mediated inhibition, PD-1 or PD-L1 blockade may not sufficiently unleash CD226 activity due to the presence of TIGIT. Conversely, anti-TIGIT alone does not release CD226 and CD28 from PD-1-mediated inhibition. Given the expression pattern of CD226 and CD28 on CD8^+^ T cell subsets that likely contribute to a robust response against tumors, optimal activation of the full repertoire of tumor-reactive CD8^+^ T cells is thus likely to require the coordinate inhibition of both TIGIT and PD-1. Although this defines a key new concept in understanding checkpoint inhibitors in cancer, we must note that TIGIT may also have additional roles in the anti-tumor response that may be CD226 independent, as evidenced by some single activity of anti-TIGIT mAb in CD226-deficient mice and when anti-TIGIT mAb is used in the preventative setting. It is possible that CD96 may play a compensatory costimulatory role in the absence of CD226 on CD8^+^ T cells (Chiang et al., 2020) or that TIGIT:PVR interaction triggers back signaling through the ITIM-containing PVR to modulate the functions of the target cell. TIGIT is also expressed by Treg cells and NK cells, and blockade of TIGIT may have modulatory effects. Moreover, there are several reports that anti-TIGIT antibodies, via an effector competent Fc domain, may activate myeloid cells during the act of antigen presentation to CD8 T cells (Dixon et al., 2018; Waight et al., 2018; Yang et al., 2020). The continued combination of data from both clinical and preclinical studies will be required to fully appreciate underlying mechanisms so that therapeutic approaches can be optimized in a systematic and rational fashion.

### Limitations of the study

We showed that TILs in human NSCLC tumors differentially expressed CD226 and CD28, indicating that activation of the full repertoire of TILs would require dual blockade of TIGIT and PD-1. While the association of CD226 expression with clinical response to atezolizumab was demonstrated, it remains to be determined whether CD226 expression is also associated with clinical benefit in patients treated with the combination of atezolizumab plus anti-TIGIT mAb tiragolumab. Currently this combination is being evaluated in a number of phase 3 studies in various indications. As these studies mature and biomarker data are collected, this question can be addressed.

Human TILs included a population of CD28^+^CD226^+^ cells that appeared to be poised for expansion, and these may be likely targets of dual blockade. In mice, however, the CD28^+^CD226^+^ TILs are exceedingly rare, making correlative analysis difficult. Given their differences in number, it is also difficult to make the assumption that the mouse double-positive TILs play the same role in tumor immunity as the analogous population in humans. Determination of whether the “on treatment” effects observed in mouse tumor models are recapitulated in human cancer patients would require matched blood and tumor biopsies, which may be quite challenging.

While our work extended the understanding of CD226 regulation by PD-1 and TIGIT from cell-free liposomal reconstitution systems to intact cells, further investigations in primary cells are warranted. This may be important in the context of TILs expressing varying levels of CD28, CD226, PD-1, and TIGIT, and myeloid or tumor cells expressing PD-L1 and PVR. In addition to CD8^+^ T cells, the interplay of these receptors on other immune cells such as NK cells and Treg cells should be further explored.

## STAR★METHODS

Detailed methods are provided in the online version of this paper and include the following:

### RESOURCE AVAILABILITY

#### Lead contact

Further information and requests for resources and reagents should be directed to and will be managed by the lead contact, Ira Mellman (mellman.ira@gene.com).

#### Materials availability

Cell lines, mouse lines and unique reagents including antibodies, oligonucleotides and recombinant DNA may be available upon request and may be subject to approval of a Material Transfer Agreement. For information regarding Material Transfer Agreements, please refer to the following: https://www.gene.com/scientists/mta.

#### Data and code availability

The accession number for the scRNaseq data reported in this paper is European Genome-phenome Archive (https://ega-archive.org/ega/): EGAS00001003993 and EGAS00001003994, and datasets EGAD00001005464 and EGAD00001005465. There are restrictions to the availability of bulk RNaseq datasets from patients enrolled in BIRCH, OAK and POPLAR clinical trials due to the informed consent signed by patients, but normalized expression matrices for specific genes may be made available under specific written request and review by the lead contact.

### EXPERIMENTAL MODEL AND SUBJECT DETAILS

#### Mice

BALB/c mice were purchased from the Jackson Laboratory. *Cd226*^−/−^ mice on BALB/cAnN background were previously described (Du et al., 2018). All mice were housed and maintained at Genentech in accordance with American Association of Laboratory Animal Care guidelines. All experimental animal studies were conducted under the approval of the Institutional Animal Care and Use Committees of Genentech Lab Animal Research and were performed in an Association for the Assessment and Accreditation of Laboratory Animal Care (AAALAC)-accredited facility.

#### Syngeneic tumor studies

CT26 tumor studies were performed by inoculating age-matched 6–8 week old female *Cd226*^−/−^ or WT littermate mice with a subcutaneous injection of 0.1 × 10^6^ CT26 cells in 100 μl HBSS+matrigel. For treatment with anti-PD-1 or anti-TIGIT mAb, treatment was initiated one day following tumor inoculation. Mice were treated with 10 mg/kg isotype control, anti-PD-1.mIgG2a LALAPG mAb (GNE 9899) or anti-TIGIT mAb (clone 10A7) three times a week for three weeks, administered i.v. first dose and subsequently i.p. Tumor volumes were measured and calculated twice per week using the modified ellipsoid formula: ½ × (length × width^2^). Animals bearing tumors exceeding 2,000 mm^3^ or showing ulceration were euthanized following approved protocols.

#### Cell lines

CT26 and B16-F10 cell lines (obtained from external vendor such as ATCC) were maintained at a dedicated internal cell line facility and tested to be mycoplasma-free. CT26 cells were cultured in RPMI 1640 media supplemented with 10% FBS and 100 U/mL penicillin/100 μg/mL streptomycin; B16-F10 cells were cultured in DMEM media supplemented with 10% FBS and 100 U/mL penicillin/100 μg/mL streptomycin, and grown in a 37°C humidified, 5% CO_2_ incubator. The B16-F10 CD155/CD112 double knockout line (referred to as PVR^−/−^PVRL2^−/−^ B16F10) has been previously described (Du et al., 2018). Jurkat E6.1 cells were obtained from Dr. Arthur Weiss (University of California San Francisco). HEK293T cells and Raji B cells were obtained from Dr. Ronald Vale (University of California San Francisco). HEK293T cells were maintained in DMEM medium (Genesee Scientific, #25-501) supplemented with 10% fetal bovine serum, 100 U/mL of Penicillin, and 100 μg/mL of Streptomycin at 37°C / 5% CO_2_. Jurkat and Raji cells were maintained in RPMI-1640 medium (Corning, #10-041-CM) supplemented with 10% fetal bovine serum, 100 U/mL of Penicillin, and 100 μg/mL of Streptomycin) at 37°C / 5% CO_2_.

#### Human subjects

Fresh tumor samples and matched adjacent non-cancerous tissues were procured from a commercial vendor (Discovery Life Sciences) as part of adult patients with NSCLC undergoing surgical resection. Patient sample information such as age, gender, ethnicity, tumor stage, tumor histology subtype (if known), tumor area category (if known), extent of lymph node spread (if known) and metastatic status (if known) is provided in Table S1. We complied with all ethical standards of the Roche Ethics Committee. Informed consent was obtained from all sampled individuals.

### METHOD DETAILS

#### Analyses of clinical trial data

Analyses were conducted using data from the POPLAR or OAK randomized, open-label studies of atezolizumab versus docetaxel in patients with NSCLC who progressed during or following prior platinum chemotherapy, or from the BIRCH study where all patients were treated with atezolizumab without any comparison treatment group. Full details on the protocols, consort diagrams, and study results have been previously described (Fehrenbacher et al., 2016; Peters et al., 2017; Rittmeyer et al., 2017). Progression-free survival (PFS) and overall survival (OS) hazard ratios were computed for each gene by dichotomizing the expression of that gene based on the value being higher or lower than the median, then fitting a Cox proportional hazards model to the censored PFS or OS survival times using the dichotomized variable to determine hazard ratio and two-sided p value. PFS was the time between the date of randomization/first atezolizumab dose and the date of first documented disease progression or death, which occurred first. Disease progression was determined based on investigator assessment per RECIST v1.1. OS was the time between the date of randomization or first atezolizumab dose and death due to any cause.

#### Human tumor processing

Fresh surgical tumor samples were shipped overnight to our institution and processed immediately upon arrival. Tumor tissues were digested using collagenase D (0.5 mg/mL) (MilliporeSigma, St. Louis, MO) and DNase (0.1 mg/mL) (MilliporeSigma, St. Louis, MO) for 15 min in a 37°C rotating incubator. Tissue samples were then subjected to mechanical dissociation using a gentleMACS Octo Dissociator (Miltenyi Biotec), followed by an additional incubation at 37°C for 10 min. Samples were filtered and washed prior to downstream applications. Dissociated cell suspensions were either directly stained for mass cytometry analysis or enriched for CD45^+^ or CD3^+^ T cells by flow cytometry for use in single-cell RNA gene expression.

#### Staining of healthy reference PBMC sample

Washed PBMCs were resuspended in an appropriate volume of PBS to obtain a cell concentration of 10^7^ cells/mL. Cells were incubated with a viability reagent, Cell-ID Cisplatin (Fluidigm, South San Francisco, CA) at a final concentration of 5 μM for 5 min on ice. Cisplatin was quenched by washing once with 5x volume of MaxPar® Cell Staining Buffer (Fluidigm, South San Francisco, CA) and centrifuged at 300 × g, then resuspended to a final concentration of 30 million cells/mL in staining buffer. To start antibody labeling, 3 million cells were transferred to Falcon® 5 mL 12 × 75 mm tubes (Corning, Corning, NY) and incubated with 5 μL of Human TruStain FcX (BioLegend, San Diego, CA) for 10 min on ice to block Fc receptor binding. Healthy PBMCs were stained with CD45-198Pt (inhouse conjugation of Clone HI30, Biolegend, San Diego, CA) as a reference to incorporate into each tumor sample as our internal reference control and tumor cells were stained with CD45-89Y (Fluidigm, South San Francisco, CA) for 30 min on ice. Cells were washed twice with 4 mL cell staining buffer before being prepared for surface staining.

#### Staining of cells for mass cytometry analysis

Differentially labeled CD45^+^ cell samples were combined together as one sample. 20% of CD45-198Pt labeled healthy PBMCs was incorporated into 3 million tumor cells labeled with CD45-89Y to a Falcon® 5 mL 12 × 75 mm tube (Corning, NY). A healthy PBMC sample labeled with only CD45-198Pt was also used as a reference control sample. A master surface antibody cocktail with all metal-conjugated antibodies (50 μL of total staining reagent volume) was added to samples for cell staining and incubated for 30 min on ice. A list of the metal conjugated mAbs used in these studies is in Table S2. Cells were washed once with 4 mL of cell staining buffer before being prepared for intracellular staining by using the FoxP3 Staining Buffer Set (Affymetrix, eBioscience, San Diego, CA). Cells were then resuspended in 1 mL of fixation/permeabilization solution for 45 min on ice. After incubation, cells were washed with 3 mL of permeabilization buffer centrifuged at 800 × g for 5 min and resuspended in 50 μL of permeabilization buffer. Cells were then stained for intracellular markers by addition of 50 μL master intracellular antibody cocktail for 30 min on ice. Lastly, the cells were washed with 4 mL cell staining buffer and fixed in a 1 mL solution overnight at 4°C containing Cell-ID Intercalator-Ir in 1.6% EMS paraformaldehyde (Electron Microscopy Sciences, Hatfield, PA) diluted with PBS. For NSCLC6, an antibody detecting CD39 (Biolegend, Clone A1) was also included in the staining panel.

#### Acquisition on CyTOF® instrument

After overnight fixation, cells were washed with 3 mL of MaxPar® cell staining buffer and centrifugated at 800 × g for 5 min. After aspiration of the wash buffer and re-suspension of the cell-pellet, another round of wash was performed with 4 mL of MaxPar® Water (Fluidigm, South San Francisco, California). Cells were resuspended in 1mL of MaxPar® Water and counted. After obtaining cell counts, 3 mL of MaxPar® Water was added and cells were pelleted one final time prior to instrument acquisition. Before introduction into the Helios CyTOF® System (Fluidigm, South San Francisco, California), pelleted cells were resuspended with 1X MaxPar® Water containing EQ Four Element Calibration Beads (Fluidigm, South San Francisco, CA) then filtered using a 12 × 75 mm tube with a 35 μm nylon mesh cell-strainer cap (Corning, Corning, NY).

#### Data processing and analysis of CyTOF® data

All FCS files from each indication were normalized together using the MATLAB® (MathWorks®, Natick, MA) normalizer and analyzed using FlowJo® software (Flowjo, Ashland, OR).

#### Aggregated UMAP analysis for mass cytometry data

Protein marker expression intensities from mass cytometry analysis were aggregated from multiple samples and transformed using the inverse hyperbolic sine function. Dimensionality reduction was applied to the transformed expression matrix using the UMAP package in R with the following default parameters: min dist: 0.1, n_neighbors: 15, n_components: 2, and metric: euclidean. For each aggregated UMAP analysis, individual samples were downsampled to equal cell numbers as following: 8000 cells per sample in CD45^+^ and CD8^+^ populations for the aggregated NSCLC analysis and ~7,500 cells per sample in the analysis of the representative tumor and adjacent tissue NSCLC sample. UMAP components were appended to “.fcs” files as additional channels for integration with manual gating analysis in FlowJo (Ashland, OR).

#### Analysis of scRNA-seq data for combined CD8^+^ T cells

CD8^+^ T cells from the dataset from (Wu et al., 2020) (available from the NCBI Gene Expression Omnibus as GSE139555) were isolated by taking single cells that were identified in that paper as having a cluster of 8.1-Teff, 8.2-Tem, 8.3a-Trm, 8.3b-Trm, 8.3c-Trm, 8.4-Chrom, 8.5-Mitosis, or 8.6-KLRB1. The single-cell RNA-seq (scRNA-seq) data were analyzed using the Seurat package (version 3.2.0) in the statistical program R (version 4.0.0). The CD8^+^ data within each tumor, NAT, and blood sample were normalized using the SCTransform function, and then combined within each patient using the FindIntegrationAnchors and IntegrateData functions, all with default parameters. The scRNA-seq data were then combined across all 6 patients using the FindIntegrationAnchors and the IntegrateData functions, with the features to integrate parameter set to be the intersection of the integration anchors over all patients.

The combined dataset was then transformed into a common coordinate space using the ScaleData, RunPCA, and RunUMAP functions, all with default parameters. The single cells were clustered using the FindNeighbors and FindClusters function with a resolution of 0.6, obtained by trial and error to yield clusters that matched most closely those from the original paper. A correspondence between the original clusters and new ones was obtained by plotting both sets of clusters onto the UMAP plot, showing that the original 8.2-Tem cluster divided into two clusters, labeled 8.2a-Tem and 8.2b-Tem, and that the original 8.4-Chrom and 8.6-KLRB1 clusters were combined into a single cluster, labeled 8.6-KLRB1. Biomarkers indicated that the 8.2a-Tem had greater expression of JUN and FOS, suggesting they represented activated T cells relative to 8.2b-Tem.

UMAP plots of individual genes and combination of genes were generated using the plot command in R, with all single cells plotted in gray as a background, and those single cells having a non-zero count for each gene plotted in a gradation of colors as a foreground. The gradation of colors was determined by the expression of the combination of genes, where each gene expression was first converted to a fractional expression by dividing its count by the total number of counts for that cell yielding a counts-per-million (cpm) statistic, and then to a Z-score, by subtracting its mean cpm and dividing by its standard deviation of cpm. The gene expression of a combination of genes was the sum of the individual gene Z-scores, and the gradation of colors was determined by the quantile of the expression among all single cells in the foreground. In all cases, the order of points plotted was randomized to avoid giving preference to any individual sample or patient. Barplots of PD-1, TIGIT, CD28, and CD226 expression and their combination were generated using the barplot command in R on a 2-dimensional table containing counts of cells expressing all of the given genes in each of the clusters, where expression was determined by a non-zero count for the given gene.

#### *In vitro* T cell cytotoxicity assay

Real-time cell electronic sensing using the xCELLigence RTCA MP system (ACEA Biosciences, San Diego, CA) was performed to assess cytotoxicity as previously described (Du et al., 2018). Briefly, OT-I cells were used as effector cells against B16-F10 or PVR^−/−^PVRL2^−/−^ B16F10 target cells. 10 × 10^6^ effector cells in volume of 5 mL complete RPMI media (cRPMI, RPMI 1640 supplemented with 10% FBS, 2 mM L-glutamine, 2 μM 2-mercaptoethanol, 1 mM sodium pyruvate, 100 U/mL penicillin and 100 μg/mL streptomycin) were pre-activated with 1 μg/mL SIINFEKL peptide for 3 days. Cells were then harvested, washed twice, then incubated for 3 days. On the day of the assay, target cells were preincubated with 1 μg/mL SIINFEKL peptide for 2 h at 37°C and 1 × 10^5^ target cells in 100 μl cRPMI were added to wells of the E-Plate VIEW 96 plate to allow loading of antigen and adherence of target cells. Effector cells harvested after the resting period were adjusted to a concentration of 4 × 10^6^ cells/mL in cRPMI. 100 μl effector cells were added to wells containing target cells. Negative control wells were target cells without effector cells. After addition of effector cells, Cell Index (CI) was measured every 10 min in real time. CI at each time point was normalized against CI at the time of effector cell addition, and cytotoxicity was calculated as % cytotoxicity = (normalized CI_no effector_ − normalized Ci_effector_)/normalized CI_no effector_ × 100.

#### Jurkat:Raji coculture assay

Each gene of interest was introduced into Jurkat and Raji cells via lentiviral transduction, as described previously (Xu et al., 2020). Briefly, each cDNA was cloned into a pHR vector backbone, and co-transfected with pMD2.G and psPAX2 packaging plasmids into HEK293T cells using polyethylenimine (PEI) (Fisher Scientific, #NC1014320). Lentiviruses were harvested at 60–72 h post-transfection. Jurkat and Raji cells were spin-infected at 35°C, 1000 × g for 30 min, and incubated at 37°C, 5% CO2 overnight. Raji (PVR-3HA^Hi^) and Raji (PVR^+^, PD-L1-TagBFP) cells were generated by transducing with PVR-3HA alone or plus PD-L1-TagBFP to WT Raji cells. Similarly, Jurkat (CD226-mGFP), Jurkat (CD226-mGFP, TIGIT^WT^-mCherry), Jurkat (CD226-mGFP, TIGIT^ΔICD^-mCherry), Jurkat (CD226-mGFP, TIGIT^FF^-mCherry), Jurkat (CD226-mGFP, PD-1^WT^ -mApple), Jurkat (CD226-mGFP, PD-1^ΔICD^-mApple) and Jurkat (CD226-mGFP, TIGIT-mCherry, PD-1-mApple) were generated by transducing WT Jurkat cells with a pHR plasmid encoding the fusion genes, driven by the SFFV promoter. Raji (PVR-3HA^Lo^) and Raji (PVR-TagBFP^Lo^) cells were generated by transducing WT Jurkat cells with a pHR plasmid encoding the fusion gene under the control of the dSV40 promoter. Raji cell lines were further sorted for PVR or PD-L1 surface expression using a FACSAria Fusion cell sorter (BD Biosciences) after staining with PE anti-PVR (BioLegend, #337609) or APC anti-PD-L1 (BioLegend, #393609).

Cell surface expression of proteins of interest on transduced Raji or Jurkat cells was confirmed by flow cytometry. Flow cytometry was conducted in an LSRFortessa cell analyzer (BD Biosciences) and data analyzed using FlowJo (FlowJo, LLC). Raji or Jurkat cells were washed with PBS and analyzed after staining with PE anti-PVR (BioLegend, #337609), APC anti-PD-L1 (BioLegend, #393609), PE anti-CD226 (BioLegend, #338305), PE anti-TIGIT (BioLegend, #372703), or PE anti-PD-1 (BioLegend, #329916), respectively.

For examining receptor phosphorylation, Jurkat cells were starved in serum free RPMI medium at 37°C for 3 h prior to coculture to decrease tonic phosphorylation. Raji B cells were pre-incubated with 30 ng/mL SEE in RPMI medium for 30 min at 37°C. Afterward, 1 × 10^6^ SEE-loaded Raji B cells and 2 × 10^6^ Jurkat T cells were precooled on ice and mixed in a 96-well plate, which was then centrifuged at 300 × g for 1 min at 4°C to initiate cell-cell contact, and immediately transferred to a 37°C water bath. The reactions were terminated with lysis buffer (50 mM HEPES-NaOH, pH 7.4, 150 mM NaCl, 1% NP-40, 5% glycerol, 1 mM PMSF, 10 mM Na_3_VO_4_, 10 mM NaF) at indicated time points. CD226-mGFP was immunoprecipitated from the lysate using GFP-Trap (Chromotek, #gta-20). TIGIT-mCherry was immunoprecipitated from the lysates using protein G Dynabeads (ThermoFisher Scientific, #10004D) coupled with an anti-mCherry antibody (GeneTex, GTX128508). Equal fractions of the IP samples were subjected to SDS-PAGE and immunoblotted with indicated antibodies.

#### Confocal microscopy

4 × 10^5^ Raji B cells were pulsed with 30 ng/mL SEE in RPMI medium for 30 min at 37°C before mixing with 4 × 10^5^ Jurkat T cells. Cell mixtures were centrifuged at 300 × g for 1 min to initiate cell-cell contact, and immediately transferred to a 37°C water bath for 5 min. The cells were fixed with equal volume of RPMI medium containing 2% PFA and loaded into a 96-well glass-bottom plate for confocal microscopy assays. Images were acquired with a Nikon spinning disk confocal microscope, then processed and quantified using ImageJ. Interface enrichment indices of CD226-mGFP on Jurkat:Raji conjugated cells were obtained through dividing the fluorescence density at the interface by the fluorescence density of the cell membrane excluding the interface. The conjugated area between a Jurkat cell and a Raji cell on the DIC images was defined as their interface. Fluorescence density was calculated as total fluorescence intensity divided by area.

#### Liposome reconstitution and FRET assays

For liposome FRET assays, human TIGIT^ICD^ (aa 192–244), PD-1^ICD^ (aa 194–288) were expressed with an N-terminal His_10_ tag in *Escherichia coli* using the pET28A vector, then purified using the Ni-NTA agarose (ThermoFisher Scientific, #88223), and eluted with HEPES buffered saline (HBS) (50 mM HEPES-NaOH, pH 7.5, 150 mM NaCl) containing 500 mM imidazole. His_10_ tagged human protein tyrosine kinase Lck^G2A^ and Fyn (aa 7–536) were expressed in the Bac-to-Bac baculovirus system (Hui and Vale, 2014).

SAP, GRB2, Shp1^tSH2^, Shp2^tSH2^, SHIP1, P50α and ZAP70^tSH2^ proteins were fused with an N-terminal GST tag followed by a PreScission recognition sequence (LEVLFQGP) and a SNAP-tag. All proteins were expressed in *Escherichia coli* using the pGEX6p-2 vector except SHIP1 which was expressed in the Bac-to-Bac baculovirus system using the pFastBac vector. Proteins were purified using Glutathione Agarose, and eluted with HBS buffer containing 20 units/mL 3C protease to remove the GST tag. All affinity-purified proteins were subjected to gel filtration chromatography using HBS buffer containing 10% glycerol and 1 mM TCEP. The monomer fractions were pooled, snap frozen and stored at − 80°C in small aliquots.

Large unilamellar liposomes consisting of 79.7% POPC, 10% POPS, 10% DGS-NTA-Ni and 0.3% Rhodamine-PE were generated by extrusion as described (Hui and Vale, 2014). Briefly, desired lipids were mixed in chloroform, dried under a nitrogen stream and subsequently in a vacuum container for 1 h. Lipid film was resuspended in 1x kinase buffer (50 mM HEPES-NaOH, pH 7.4, 150 mM NaCl, 10 mM MgCl_2_) and extruded for 20 times through a pair of polycarbonate filters containing pores of 200 nm diameter. For Figures 5F, G and S6C, D, 2.8 nM liposomes were incubated with indicated concentrations of His_10_-Fyn^AA7−536^, 300 nM receptor tails (His_10_-TIGIT^ICD^ or His_10_-PD-1^ICD^), 100 nM non-His-tagged SH2 containing proteins (SC505*SAP, SC505*GRB2, SC505*Shp1^tSH2^, SC505*Shp2^tSH2^, SC505*SHIP1, SC505*ZAP70^tSH2^ or SC505*P50α), in 1 × kinase buffer supplemented with 10 mM sodium vanadate, 0.5 mg/mL BSA and 1 mM TCEP, in a 96-well solid white microplate (Greiner Bio-One, 655075). The fluorescence of energy donor SC505 was monitored in real time using a plate reader (Tecan Spark 20) with 504-nm excitation and 540-nm emission. Following 40 min incubation, ATP was added to a final concentration of 1 mM and SC505 fluorescence monitored for an additional 1 h. Data were normalized by the mean fluorescence intensity of the last twenty data points before the addition of ATP and plotted with GraphPad Prism 5.0. All liposome-based recruitment assays were conducted at room temperature.

For Figure S6A, kinase titrations with His_10_-TIGIT^ICD^ or His_10_-PD-1^ICD^ were conducted under the same conditions as that of liposome-based recruitment assays except (1) using different concentrations of kinase and (2) in the absence of SC505 labeled SH2 proteins. The reactions were stopped by the addition of an equal volume of 2 × SDS sample buffer at 30 min or 150 min and subjected to SDS-PAGE and immnunoblotted with indicated antibodies.

#### Jurkat:SLB imaging assay

To form SLB, a glass bottom 96-well plate (Cellvis, P96-1.5H-N) was incubated with 2.5% Hellmanex III (Hëlma Analytics, Z805939) overnight on a 50°C heat pad before thoroughly rinsed with ddH2O and sealed. The desired wells were washed twice with 6 M NaOH, and thrice with 500 μL ddH2O and 1x PBS respectively. Small unilamellar vesicles (SUVs, consisting of 97.5% POPC, 2% biotin-DPPE, 2% DGS-NTA-Ni and 0.5% PEG5000-PE) were prepared as described previously (Hui et al., 2017) and added to the cleaned wells containing 200 μL 1x PBS, and incubated for 120 min at 50°C, followed by 30 min at RT to induce SLB formation. The SLBs were rinsed with wash buffer (1x PBS containing 0.1% BSA) and overlaid with a solution containing 1 μg/mL streptavidin, 2 nM His-tagged human PVR extracellular domain (ECD), 1 nM His-tagged human PD-L1^ECD^, and 3 nM His-tagged human ICAM-1^ECD^ at RT for 1.5 h. Afterward, the SLBs were rinsed with wash buffer and further incubated with 5 μg/mL biotin anti-human-CD3ε (clone Okt3) at RT for 45 min, followed by three rinses with wash buffer and three rinses with imaging buffer (20 mM HEPES pH 7.5, 137 mM NaCl, 5 mM KCl, 1 mM CaCl_2_, 2 mM MgCl_2_, 0.7 mM Na_2_HPO_4_, 6 mM D-Glucose). Indicated Jurkat cells were harvested via centrifugation at 200 × g for 4 min, incubated with 40 μg/mL anti-TIGIT antibody (clone 10A7) or pembrolizumab for 30 min at RT. After washing both SLBs and Jurkat cells twice with 1x PBS and once with imaging buffer, cells were overlaid onto functionalized SLBs for 10 min at 37°C before fixing with 2% PFA in PBS for another 10 min. TIRF images were acquired at room temperature using a Nikon Eclipse Ti microscope equipped with a 100 × Apo TIRF 1.49 NA objective and an Andor iXon Ultra 897 EMCCD camera, controlled by the Micro-Manager software (Edelstein et al., 2014). Fiji was used to quantify the fluorescent intensities of TIGIT or PD-1 in CD226 microclusters (Schindelin et al., 2012). Mask images identifying the area of CD226 microclusters were generated by applying the “subtract background” command to CD226 (mGFP) images using the default setting (Xu et al., 2021). The fluorescent signals of CD226 (mGFP), TIGIT (mCherry), or PD-1 (mApple) in the masked overlaid images were measured, and used to calculate the CD226 FI/TIGIT FI or CD226 FI/PD-1 FI ratio for each cell.

#### GST pull down

For Figure S6A, D, soluble GST-tagged baits (TIGIT^ICD^ or PD-1^ICD^, 10 μg each) were phosphorylated by 0.2 μg His_10_-Lck^G2A^ and 0.2 μg His_10_-Fyn^aa7−536^ at room temperature in the presence of 1 mM ATP for 3 h, then mixed with clear cell lysate of 3 × 10^7^ Jurkat cells pre-stimulated by 1.5 × 10^7^ SEE-pulsed Raji cells, as described (Xu et al., 2020). After an incubation at 4°C for 3 h, 100 μL glutathione agarose resin (GoldBio, #G-250) was added into the mixture and rotated for another 1 h at 4°C. Resin was then rinsed 4 times with 1 mL wash buffer (20 mM HEPES-NaOH, pH 7.4, 50 mM NaCl, 0.1% NP-40, 1 mM EDTA, 10 mM Na_3_VO_4_, 10 mM NaF), and proteins eluted by 1 × SDS sample buffer at 95°C for 10 min and subjected to SDS-PAGE.

#### Sample processing for mass spectrometry

The samples were in-gel digested and analyzed using a nanoLC-MS/MS as previously described (Xu et al., 2020)[REMOVED HYPERLINK FIELD]. Briefly, the gel pieces were reduced with 10 mM DTT (Acros Organics, #16568-0050) at 56°C for 30 min, then alkylated in dark with 50 mM IAA (MP Biomedicals, #100351) for 30 min at RT before digesting overnight with Trypsin (Promega, #V511A) at a ratio of 1:100 (enzyme: substrate) in 50 mM ammonium bicarbonate (Sigma-Aldrich, #5330050050) buffer at 37°C. The supernatant of digested sample was transferred to 1.5 mL tube, then peptides were extracted from the gel pieces using 10% formic acid and 100% acetonitrile (ACN) sequentially before combining with the above supernatant and dried in a speed-vac. Dried peptides were then resuspended in 10 uL reconstitution solution (5% Acetonitrile/5% Formic acid) and subjected to MS analysis.

#### Liquid chromatography-tandem mass spectrometry (LC-MS/MS)

Peptide samples were analyzed in duplicate by nLC-MS/MS, an EASY-nLC 1000 (Thermo Scientific) chromatography system coupled with a Q-Exactive mass spectrometer (Thermo Scientific, San Jose, CA). Peptides were first separated by reverse-phase chromatography using a C18 reverse-phase resin (ReproSil-pur 120 C18-AQ, 1.9 μm, Dr. Maisch GmbH) packed in a fused silica microcapillary column (75 μm ID, 15 cm) using an in-line nano-flow EASY-nLC 1000 UHPLC. Peptides were eluted at a flow rate of 250 nL/min for a total running time of 120 min over 4 gradients: a 100 min 2%–30% ACN gradient, a 5 min 30%–60% ACN gradient, a 5 min 60%–95% ACN gradient, with a final 10 min step at 0% ACN. Mobile phases of all gradients contained 0.1% formic acid. MS/MS data were collected in a data-dependent fashion using a top 10 method with a full MS mass range from 400–1800 m/z, 70,000 resolution, and an AGC target of 3e6. MS2 scans were triggered when an ion intensity threshold of 1e5 was reached with a maximum injection time of 60 ms. Peptides were fragmented using a normalized collision energy setting of 25. A dynamic exclusion time of 40 s was applied and the peptide match setting was disabled. Singly charged ions, charge states above 8 and unassigned charge states were excluded. The MS/MS spectra were searched against the UniProt Human reference proteome database (downloaded Sept 20^th^, 2021) integrated with WT and signaling-deficient mutant of GST-TIGIT^ICD^ (aa 192–244) and GST-PD-1^ICD^ (aa 194–288) sequences, using SEQUEST in Proteome Discoverer 2.1 (TermoFisher Scientific) with 1% False Discovery Rate (FDR). Identified proteins were quantified through the abundance of their unique peptides. Database queries from UniProt were used to sort for SH2-containing proteins.

#### Plasmid Construction

The sequence encoding the SNAP tag were amplified by PCR from the pT8-SNAP (Cisbio) to produce a CMV-based vector pRK.FLAG.SNAP vector. hCD226 gene was PCR-amplified and cloned into this vector to express N-terminally tagged proteins. To generate hCD226-KLB TMD construct, the native TMD sequence was replaced by the TMD of hKlotho-β (KLB) of the same length encompassing TMD (-LIFLGCCFFSTLVLLLSIAIF-). For hCD226- KLB TMD-ΔICD construct, an octahistidine tag and stop codon were introduced immediately downstream of KLB TMD.

#### TR-FRET

COS7 cells were transfected with N terminus SNAP-tagged (ST) CD226 and N terminus full length or chimeric HA-TIGIT using Lipofectamine 2000 (Life Technologies) and seeded in a white 96-well plate (Costar) at 100,000 cells per well. TR-FRET experiments were conducted as reported previously (Johnston et al., 2014). Briefly, cells were labeled with 1 μM acceptor conjugated benzyl-guanine SNAP-A647 (New England Biolabs) and 2 nM anti-HA Lumi-4Tb (Cisbio). Cells were then washed and the donor emission as well as the TR-FRET signal were recorded using a CLARIOstar Plus microplate reader (BMG LabTech). The deltaR was calculated as followed: (TR-FRET signal at 665 nm / donor emission at 620 nm from labeled transfected cells) - (TR-FRET signal at 665 nm / donor emission at 620 nm from labeled mock transfected cells).

#### ELISA

COS7 cells were fixed with 4% paraformaldehyde, washed, and blocked with phosphate-buffered saline + 1% fetal calf serum (FCS). Cells were then incubated with anti-HA monoclonal antibody (clone 3F10, Roche applied science) or anti-Flag-M2 monoclonal antibody (Sigma) conjugated with horseradish peroxidase, washed and incubated with SuperSignal ELISA substrate (Pierce) for chemoluminescence detection. Signal was recorded using a CLARIOstar Plus microplate reader (BMG LabTech). Specific signal was calculated by subtracting the signal recorded on mock transfected cells.

### QUANTIFICATION AND STATISTICAL ANALYSIS

Data were analyzed using GraphPad Prism software version 9 (GraphPad, San Diego, CA). Measures between two groups were performed with a Student’s t test (two-tailed). Groups of three or more were analyzed by one-way or two-way analysis of variance (-ANOVA) followed by Tukey’s post-testing for multiple comparisons, as appropriate. *P*-values < 0.05 were considered significant. Cox proportional hazard models were determined using the coxph function in the survival package of R, followed by the summary function to obtain the hazard ratio and p value from the Wald test. For comparison of CD226 and CD28 expression in CD8^+^ T cell clusters, *p*-values were computed by a Fisher exact test on the number of cells without and with expression of the given gene within the cluster. Odds ratios are for CD226 expression relative to CD28 expression.

## Supplementary Material

1

## Figures and Tables

**KEY RESOURCES TABLE:** 

REAGENT or RESOURCE	SOURCE	IDENTIFIER

Antibodies		

See Table S2 for CyTOF reagents	This paper	N/A
Anti-PD-1 (GNE9899)	This paper	N/A
Anti-PD-1 (Pembrolizumab)	Selleck Chemicals	Cat#: A2005
Anti-TIGIT (10A7)	Yu et al., 2009	N/A
CD226 blocking antibody (DX11)	GeneTex	Cat# GTX76029; RRID:AB_377177
PE anti-human PVR (SKII.4)	BioLegend	Cat#: 337609; RRID: AB_2253258
APC anti-PD-L1 (MIH2)	BioLegend	Cat#: 393609; RRID: AB_2749926
PE anti-human CD226 (11A8)	BioLegend	Cat#: 338305; RRID: AB_2259834
PE anti-human TIGIT (A15153G)	BioLegend	Cat#: 372703; RRID: AB_2632729
Pacific Blue anti-human PD-1 (EH12.2H7)	BioLegend	Cat#: 329916; RRID: AB_2283437
Biotin anti-human CD3ε (OKT3, mouse monoclonal)	BioLegend	Cat#: 317320; RRID: AB_10916519
Anti-GFP	Invitrogen	Cat#: A-6455; RRID: AB_221570
Rabbit polyclonal anti-mCherry	GeneTex	Cat#: GTX128508; RRID: AB_2721247
Mouse monoclonal anti-phosphotyrosine (pY)	Sigma-Aldrich	Cat#: P4110; RRID: AB_477342
Mouse anti-human CD3 zeta pY142	BD Bioscience	Cat#: 558489; RRID: AB_647152
Mouse anti-FLAG M2	Sigma-Aldrich	Cat#: F3165-1MG; RRID:AB_259529
Anti-HA	Roche Applied Science	Cat#: 11867423001; RRID:AB_390918
HRP anti-His Tag Antibody	BioLegend	Cat#: 652503; RRID: AB_2734520
Rabbit polyclonal anti-GAPDH	Proteintech Group	Cat#: 10494-1-AP; RRID: AB_2263076

Biological samples		

NSCLC human sample information	This paper	Table S1

Chemicals, peptides, and recombinant proteins		

Collagenase D	Millipore-Sigma	Cat#: COLLD-RO
DNase I	Millipore-Sigma	Cat#: 10104159001
DMEM High Glu w/Gln w/o Pyr.	Thermo Fisher Scientific	Cat#: MT10017CV
RPMI 1640, w/Gln & 25 mM HEPES	Corning	Cat#: MT 10-041-CM
Fetal Bovine Serum, Heat Inactivated	Omega Scientific	Cat#: FB-02
Paraformaldehyde	Fisher Scientific	Cat#: 50980494
SEE super antigen	Toxin Technologies	Cat#: ET404
100 × Penicillin-Streptomycin	GE Healthcare	Cat#: SV30010
Polyethylenimine (PEI)	Fisher Scientific	Cat#: NC1014320
ATP	Gold Biotech	Cat#: A-081-100
1-palmitoyl-2-oleoyl-glycero-3-phosphocholine (POPC)	Avanti Polar Lipids	Cat#: 850457C
1-palmitoyl-2-oleoyl-sn-glycero-3-phospho-L-serine (POPS)	Avanti Polar Lipids	Cat#: 840034C
1,2-dioleoyl-sn-glycero-3-[(N-(5-amino-1-carboxypentyl) iminodiacetic acid) succinyl] nickel salt (DGS-NTA-Ni)	Avanti Polar Lipids	Cat#: 790404C
N-(lissamine rhodamine B sulfonyl)-1,2-dipalmitoyl-sn-glycero-3 phosphoethanolamine (Rhodamine-PE)	Avanti Polar Lipids	Cat#: 810158C
1,2-dipalmitoyl-sn-glycero-3-phosphoethanolamine-N-(biotinyl) (sodium salt)	Avanti Polar Lipids	Cat#: 870285P
1,2-dioleoyl-sn-glycero-3-phosphoethanolamine-N-[methoxy(polyethylene glycol)-5000] ammonium salt (PEG 5000-PE)	Avanti Polar Lipids	Cat#: 880230C]
GFP-Trap	Chromotek	Cat#: gta-20
imidazole	Sigma-Aldrich	Cat#: I202
Glutathione Agarose Resin	Gold Biotechnology	Cat#: G-250-50
Ni-NTA Resin	ThermoFisher	Cat#: Ni-NTA Resin
TCEP-HCl	Gold Biotechnology	Cat#: TCEP10
Protein G Dynabeads	ThermoFisher Scientific	Cat#: 10004D
Human PVR -His	Fisher Scientific	Cat#: 50-161-4026
Human PD-L1-His	Sino Biological	Cat#: 10084-H08H
Human ICAM-1-His	Sino Biological	Cat#: 10346-H08H
Streptavidin	Invitrogen	Cat#: S888
SIINFEKL peptide	AnaSpec	Cat#: AS-60193-1

Experimental models: Cell lines		

CT26	ATCC	RRID: CRL-2638
B16-F10	ATCC	RRID: CRL-6475
PVR^−/−^PVRL2^−/−^ B16-F10	Du et al., 2018	N/A
Jurkat E6.1	Provided by Dr. Arthur Weiss (University of California San Francisco)	RRID: CVCL_0065
Raji	Provided by Dr. Ronald Vale (University of California San Francisco)	RRID: CVCL_0511
HEK293T	Provided by Dr. Ronald Vale (University of California San Francisco)	RRID: CVCL_0063

Experimental models: Organisms/strains		

BALB/c	The Jackson Laboratory	N/A
CD226^−/−^ mice on BALB/c background	Du et al., 2018	N/A

Oligonucleotides		

CD226 Fwd: ggagctctcgagaattctcacgcgtATGGATTATCCTACTTTACTTTTG	This paper	N/A
CD226 Rev: gcaagcttgatatcctgcagAACTCTAGTCTTTGGTCTGC	This paper	N/A
TIGIT Fwd: tggagctctcgagaattctcacgATGCGCTGGTGTCTCC	This paper	N/A
TIGIT Rev: gcaagcttgatatcctgcagacgACCAGTCTCTGTGAAGAAGC	This paper	N/A
PD-1 Fwd: ggagctctcgagaattctcATGCAGATCCCACAGGCG	This paper	N/A
PD-1 Rev: aagcttgatatcctgcagacgcgtcaggggccaagagcagtg	This paper	N/A
PVR Fwd: ggagctctcgagaattctcacgcgtATGGCCCGAGCCATG	This paper	N/A
PVR Rev: gacccaccagatccacgcgtCCTTGTGCCCTCTGTCTGT	This paper	N/A

Recombinant DNA		

pHR-CD226-mGFP	This paper	N/A
pHR-hCD226 (Y322F)-mGFP	This paper	N/A
pHR-hCD226 (S329A)-mGFP	This paper	N/A
pHR-hCD226 (Y322F, S329A)-mGFP	This paper	N/A
pHR-TIGIT-mCherry	This paper	N/A
pHR-TIGIT (Y225F, Y231F)-mCherry	This paper	N/A
pHR-TIGIT (AA1-164)-mCherry	This paper	N/A
pHR-PD-1-mApple	This paper	N/A
pHR-PD-1 (AA1-197)-mApple	This paper	N/A
pHR-PD-1-iRFP670	This paper	N/A
pHR-PVR-3HA	This paper	N/A
pHR-SV40-PVR-3HA	This paper	N/A
pHR-SV40-PVR-tagBFP	This paper	N/A
pET28a-His10-TIGIT (AA192-244)-LPETG	This paper	N/A
pET28a-His10-PD-1 (AA194-288)-LPETG	Hui et al., 2017	N/A
pET28a-GST-TIGIT (AA192-244)-TwinStrep	This paper	N/A
pET28a-GST-TIGIT (AA192-244, Y225F, Y231F)-TwinStrep	This paper	N/A
pGEX-6P-2-GST-PreSci site-PD-1(AA194-288)	Xu et al., 2020	N/A
pGEX-6P-2-GST-PreSci site-PD-1(AA194-288, Y223F, Y248F)	Xu et al., 2020	N/A

Software and algorithms		

FlowJo v10.5.3	FlowJo, LLC.	https://www.flowjo.com
GraphPad Prism v5, v8, and v9.3.1	GraphPad Software, Inc	https://www.graphpad.com
xCELLigence RTCA MP System	Agilent	https://www.agilent.com
R (v4.0.0)	R Development Core Team, 2008	http://www.r-project.org
Seurat v3.2.0	Stuart et al., 2019	N/A
Fiji	MPI-CBG	PMID: 22743772; RRID: SCR_002285
Micro-Manager	Github	PMID: 25606571; RRID: SCR_000415

Other		

96 well glass bottom plate	Cellvis	P96-1.5H-N
Sequence data, analyses, and resources related to scRNA-seq	This paper; Wu et al., 2020	N/A
NSCLC CyTOF dataset (Repository ID: FR-FCM-Z3NA)	This paper; Banchereau et al., 2021	http://www.flowrepository.org

## Highlights

- CD226 is required for response to anti-PD-(L)1 or anti-TIGIT antibody treatment
- Tumor-infiltrating lymphocytes differentially express CD226 and CD28
- PD-1 and TIGIT impair CD226 phosphorylation through distinct mechanisms
- Convergence of PD-1 and TIGIT to inhibit CD226 provides rationale for dual blockade
