# Supplementary material for: Mechanistic convergence of the TIGIT and PD-1 inhibitory pathways necessitates co-blockade to optimize anti-tumor CD8+ T cell responses
Source: Immunity. Author manuscript; Available in PMC 2022 Jul 16. (PMC9287124; doi:10.1016/j.immuni.2022.02.005)
Supplement: 1 [file NIHMS1820604-supplement-1.pdf]

Immunity, Volume *55*

Supplemental information

Mechanistic convergence of the TIGIT and PD-1 inhibitory pathways necessitates co-blockade to optimize anti-tumor $CD8^+$ T cell responses

Karl L. Banta, Xiaozheng Xu, Avantika S. Chitre, Amelia Au-Yeung, Chikara Takahashi, William E. O'Gorman, Thomas D. Wu, Stephanie Mittman, Rafael Cubas, Laetitia Comps-Agrar, Amit Fulzele, Eric J. Bennett, Jane L. Grogan, Enfu Hui, Eugene Y. Chiang, and Ira Mellman

Mechanistic convergence of the TIGIT and PD-1 inhibitory pathways necessitates co-blockade to optimize anti-tumor CD8^{+} T cell responses

Karl L. Banta[1,5], Xiaozheng Xu[2.,5], Avantika S. Chitre[1], Amelia Au-Yeung[1], Chikara Takahashi[1], William E. O'Gorman[1], Thomas D. Wu[1], Stephanie Mittman[1], Rafael Cubas[1,3], Laetitia Comps-Agrar[1], Amit Fulzele[2], Eric J. Bennett[2], Jane L. Grogan[1,4], Enfu Hui[2,6], Eugene Y. Chiang[1,6*], and Ira Mellman[1,6,7*]

[1] Genentech, Inc., 1 DNA Way, South San Francisco, CA 94080. [2] Section of Cell & Developmental Biology, Division of Biological Sciences, University of California, San Diego, 9500 Gilman Drive, La Jolla, CA 92093, [3] Present address: Iovance, 3802 Spectrum Blvd., Tampa, FL 33612, [4] Present address: Graphite Bio, 279 E. Grand Avenue, South San Francisco, CA 94080. [5] These authors contributed equally to this work. [6] Senior authors. [7] Lead contact.

[*] Corresponding authors:
Eugene Y. Chiang, email: chiang.eugene@gene.com
Ira Mellman, email: mellman.ira@gene.com; mobile: 650-452-3894

SUPPLEMENTAL INFORMATION

Table S1. Patient characteristics and tumor metadata by individual for tissue samples analyzed by CyTOF and scRNAseq, Related to Figures 3 and 4.

Sample Code	NSCLC1	NSCLC2	NSCLC3	NSCLC4	NSCLC5	NSCLC6
Matched scRNAseq	NA	NA	NA	Y	Y	Y
Tumor Indication	NSCLC	NSCLC	NSCLC	NSCLC	NSCLC	NSCLC
Age	72	83	51	60	55	67
Gender	F	M	M	F	M	F
Ethnicity	White	White	White	White	White	White
Tumor Stage	Ia	IIa	III	I	III	Not Available
Histology Subtype	Adenocarcinoma	Squamous Cell Carcinoma	Squamous Cell Carcinoma (Keratinizing)	Squamous Cell Carcinoma (Nonkeratinizing)	Not Available	Not Available
T (Area of Cancer)	T1b	T2b	T2a	T2a	T4	Not Available
N (Lymph Node Spread)	N0	N0	N2	N0	N1	Not Available
M (Metastasis)	MX	MX	MX	MX	MX	Not Available

Table S2. Antibodies used for CyTOF panel to phenotype tumor immune cell infiltrate, Related to Figure 3.

Mass	Metal	Target	Source	Vendor	Clone	Catalog Number	Lot Number
89	Y	CD45	Fluidigm		HI30	3089003B	2291710
113	In	EpCAM	Custom	Biolegend	9C4		In-house
115	In	CD57	Custom	Biolegend	HCD57		In-house
140	Ce	EQ Beads					
141	Pr	Perforin	Custom	Abcam	B-D48		In-house
142	Nd	CCR4	Custom	R&D	205410		In-house
143	Nd	CD127/ CD137/CCR8	Fluidigm/ Custom	Biolegend	A019D5/ 4B4-1/ L263G8	3143012B	2191516 In-house
144	Nd	Granzyme B	Custom	Biolegend	GB11		In-house
145	Nd	CD4	Fluidigm		RPA-T4	3145001B	1931719
146	Nd	CD8	Fluidigm		RPA-T8	3146001B	1671716
147	Sm	CD11c	Fluidigm		Bu15	3147008B	0731704
148	Nd	CD56	Custom	Miltenyi	REA196		In-house
149	Sm	Granzyme A	Custom	Biolegend	CB9		In-house
150	Nd	CD103	Custom	Biolegend	Ber-ACT8		In-house
151	Eu	ICOS	Custom	eBioscience	ISA-3		In-house
152	Sm	CD155 PVR/ CD39	Custom	Biolegend	SKII.4/ A1		In-house
153	Eu	TIM3	Custom	R&D	344823		In-house
154	Sm	CD3	Fluidigm		UCHT1	3154003B	1351723
155	Gd	CD27	Fluidigm		L128	3155001B	1031712
156	Gd	CXCR3	Fluidigm		G025H7	3156004B	1681613
157	Gd	CD14	Custom		M5E2		In-house
158	Gd	OX40	Custom	Genentech	1A7.gr.1		In-house
159	Tb	CD226	Custom	BD	DX11		In-house
160	Gd	Tbet	Fluidigm		4B10	3160010B	1061601
161	Dy	CTLA4	Custom	Biolegend	BNI3		In-house
162	Dy	Foxp3	Fluidigm		PCH101	3162011A	0731702
163	Dy	Eomes	Custom	eBioscience	WD1928		In-house
164	Du	CD161	Fluidigm		HP-3G10	3164009B	3271610
165	Ho	CD19	Fluidigm		HIB19	3165025B	3121611
166	Er	NKG2D	Fluidigm		ON72	3166016B	0091703
167	Er	CCR7	Fluidigm		G043H7	3167009A	0241815
168	Er	Ki-67	Fluidigm		Ki-67	3168007B	0861703
169	Tm	CD25	Fluidigm		2A3	3169003B	1931713
170	Er	CD45RA	Fluidigm		HI100	3170010B	0981521
171	Yb	PD-L1	Custom	Genentech	14D3		In-house
172	Yb	CD28	Custom	Biolegend	CD28.2		In-house
173	Yb	TIGIT	Fluidigm		MBSA43	3999999-5	1461701
174	Yb	HLA-DR	Fluidigm		L243	3174001B	0041719
175	Lu	PD-1	Fluidigm		EH12.2H7	3175008B	2431709
176	Yb	CD38	Custom	Biolegend	HIT2		In-house
191	Ir	Nucleic acid	Fluidigm			201192A	2451506A
192	Pt	Cisplatin	Fluidigm			201064	0671809
193	Ir	Nucleic acid	Fluidigm			201192A	2451506A
195	Pt	Cisplatin	Fluidigm			201064	0671809
209	Bi	CD16	Fluidigm		3G8	3209002B	2031601

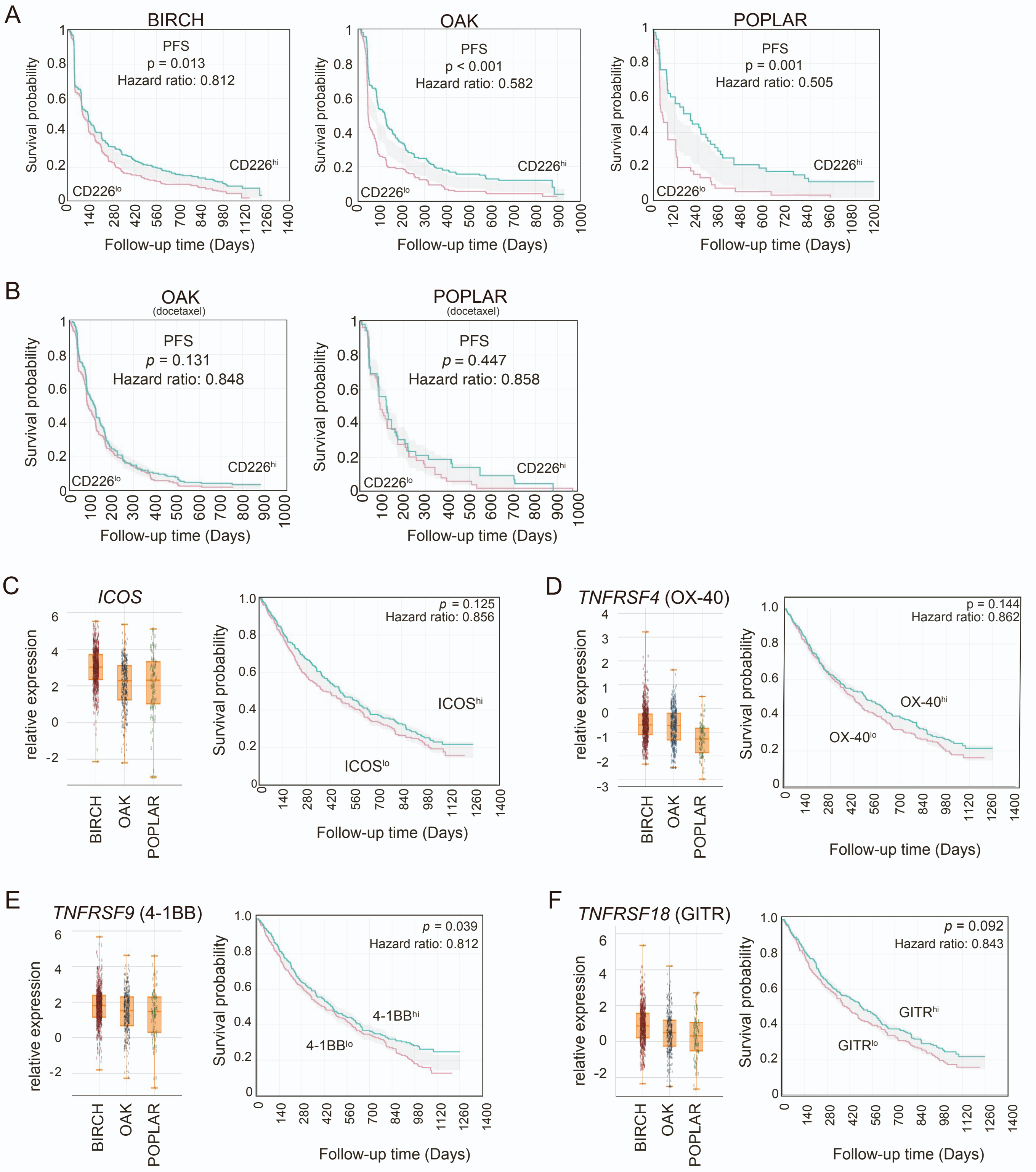

Figure S1. CD226 gene expression is associated with progression-free survival (PFS) in NSCLC with clinical response to atezolizumab but not docetaxel, and association of activating receptors with response to atezolizumab, Related to Figure 2.

Figure S1. CD226 gene expression is associated with progression-free survival (PFS) in NSCLC with clinical response to atezolizumab but not docetaxel, and association of activating receptors with response to atezolizumab, Related to Figure 2.
(A-B) Kaplan-Meier plots of PFS are shown for the atezolizumab arm (A) or docetaxel arm (B) in the indicated clinical trial. (C-F) Left panel, relative gene expression of *ICOS* (C), *TNFRSF4* encoding OX-40 (D), *TNFRSF9* encoding 4-1BB (E), and *TNFRSF18* encoding GITR (F) in BIRCH, OAK or POPLAR. Right panel, Kaplan-Meier plots of OS for indicated costimulatory markers are shown for BIRCH of the atezolizumab trials. In all plots, patients were separated on the basis of indicated gene expression into high (green line) or low (red line) expression relative to the median expression over all patients in the corresponding clinical trial. Two-sided p-values and hazard ratios from a Cox proportional hazards model are indicated for each plot.

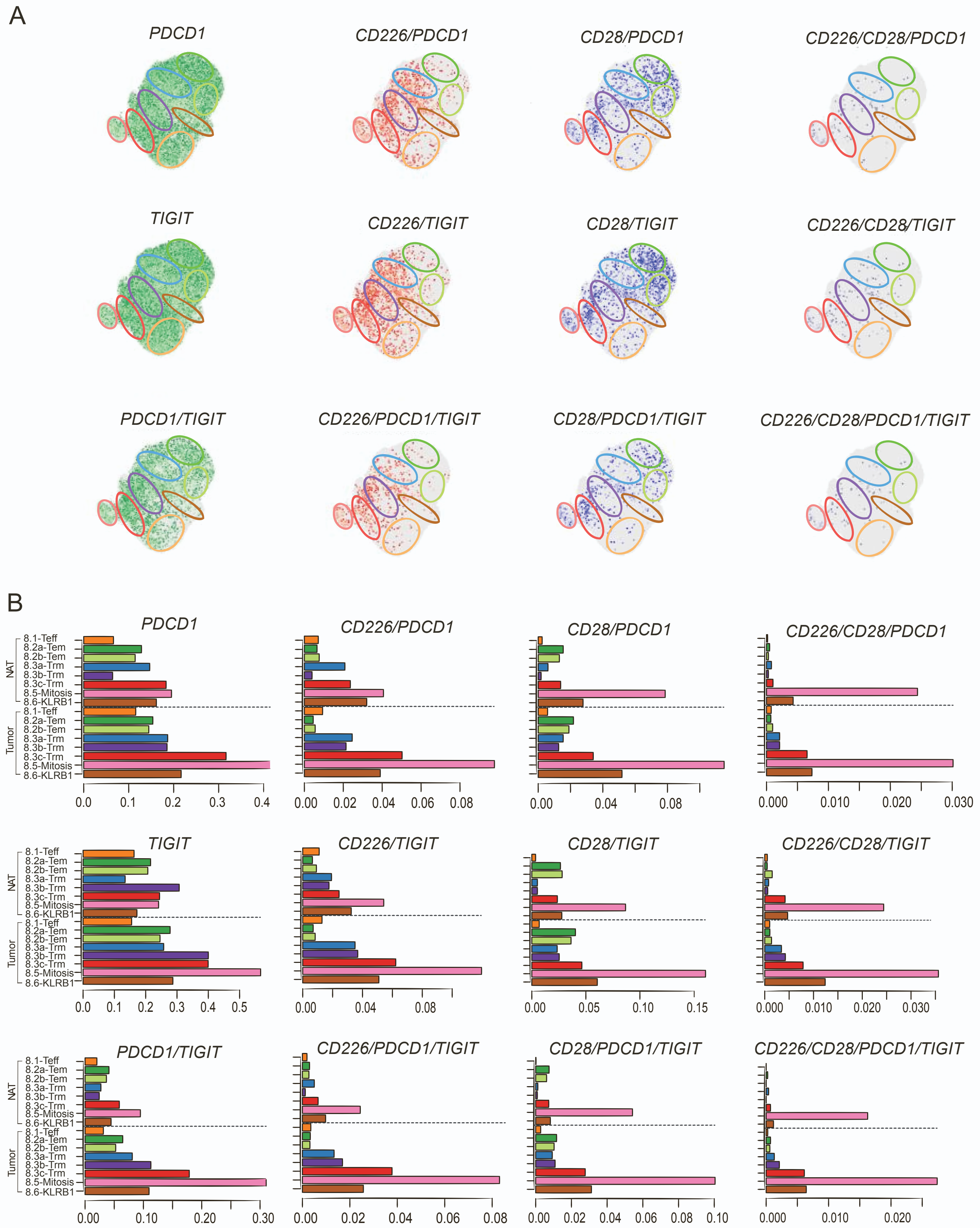

Figure S2. Expression of CD226, CD28, PD-1, and TIGIT by scRNA-seq in NSCLC tumors, Related to Figure 4.

Figure S2. Expression of CD226, CD28, PD-1, and TIGIT by scRNA-seq in NSCLC tumors, Related to Figure 4.

(A) Presence of expression for an indicated gene or gene combination of individual CD8$^+$ T cells are plotted within each UMAP overlay from Figure 4A. Labeled ovals demarcate approximate regions of the clusters. (B) Frequency of individual gene or gene combination of *CD226*, *CD28*, *PD-1,* and/or *TIGIT* within each CD8$^+$ T cell cluster are plotted by bar graph.

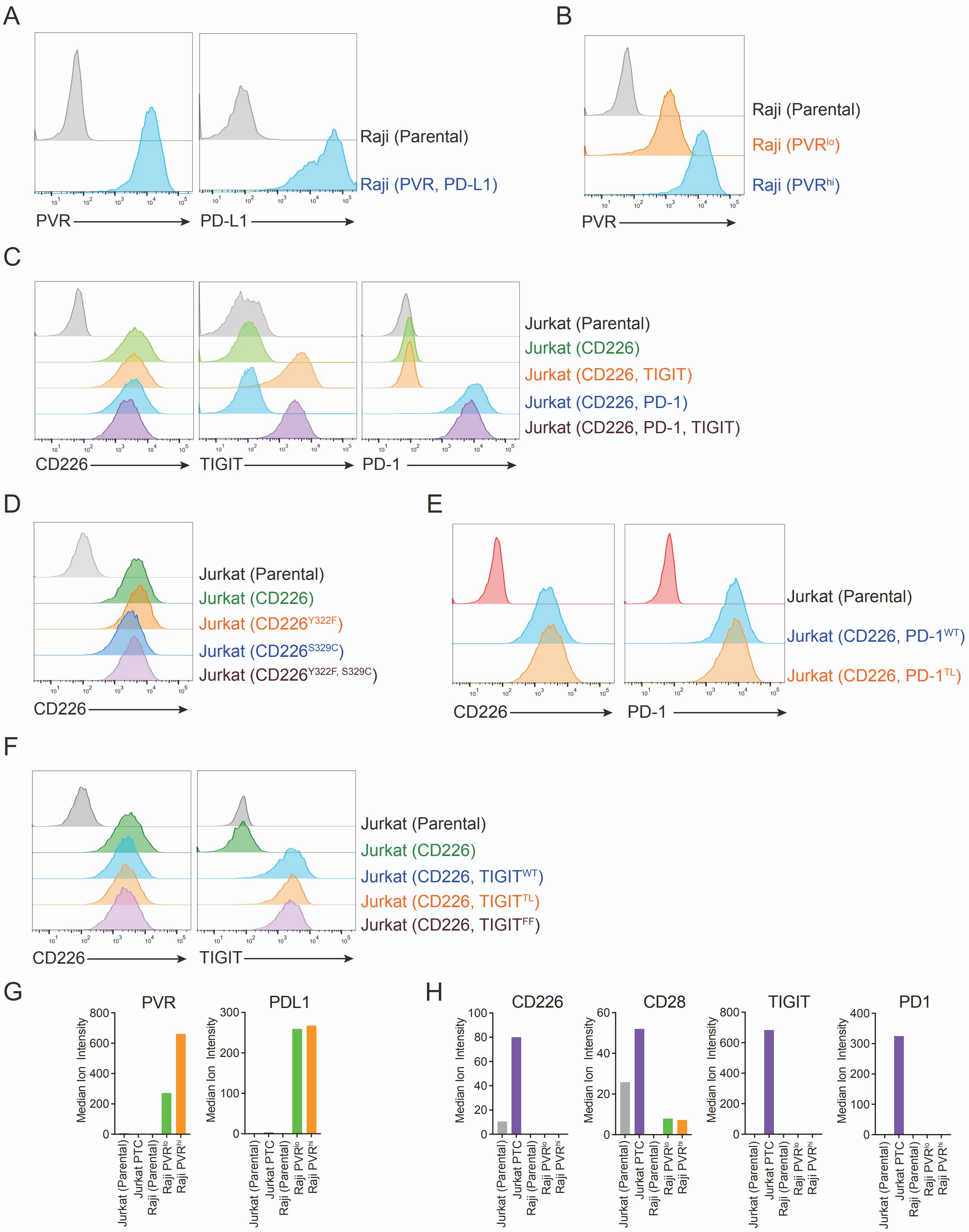

Figure S3. Receptor and ligand expression profiles on cell lines used in co-culture assays, Related to Figures 5 and 6.

Figure S3. Receptor and ligand expression profiles on cell lines used in co-culture assays, Related to Figures 5 and 6.

(A-F) Representative flow cytometry histograms showing surface expression levels of PVR, PD-L1 in Raji cells or CD226, TIGIT, and/or PD-1 in Jurkat cells. (A) Expression of PVR and PD-L1 in Raji cells. (B) Profile of PVR^{lo} and PVR^{hi} expressing Raji cells. (C) CD226, TIGIT and PD-1 expression in Jurkat cells. (D) Expression of WT CD226 or a Y322F, S329C, or both mutants within the CD226 protein in Jurkat cells. (E) Expression of PD-1 or PD-$1^{\Delta ICD}$ in Jurkat cells. (F) Co-expression of WT TIGIT or TIGIT mutant constructs with CD226 in Jurkat cells. The $TIGIT^{\Delta ICD}$ mutant is devoid of an intracellular tail, while $TIGIT^{FF}$ contains both critical tyrosine residues mutated to phenylalanine within the ICD . (G,H) CyTOF analysis of surface expression levels of the ligands PVR and PD-L1 (G) or the receptors CD226, CD28, TIGIT, and PD-1 (H) on Raji or Jurkat cell lines. Jurkat PTC denotes Jurkat cells transfected with PD-1, TIGIT and CD226.

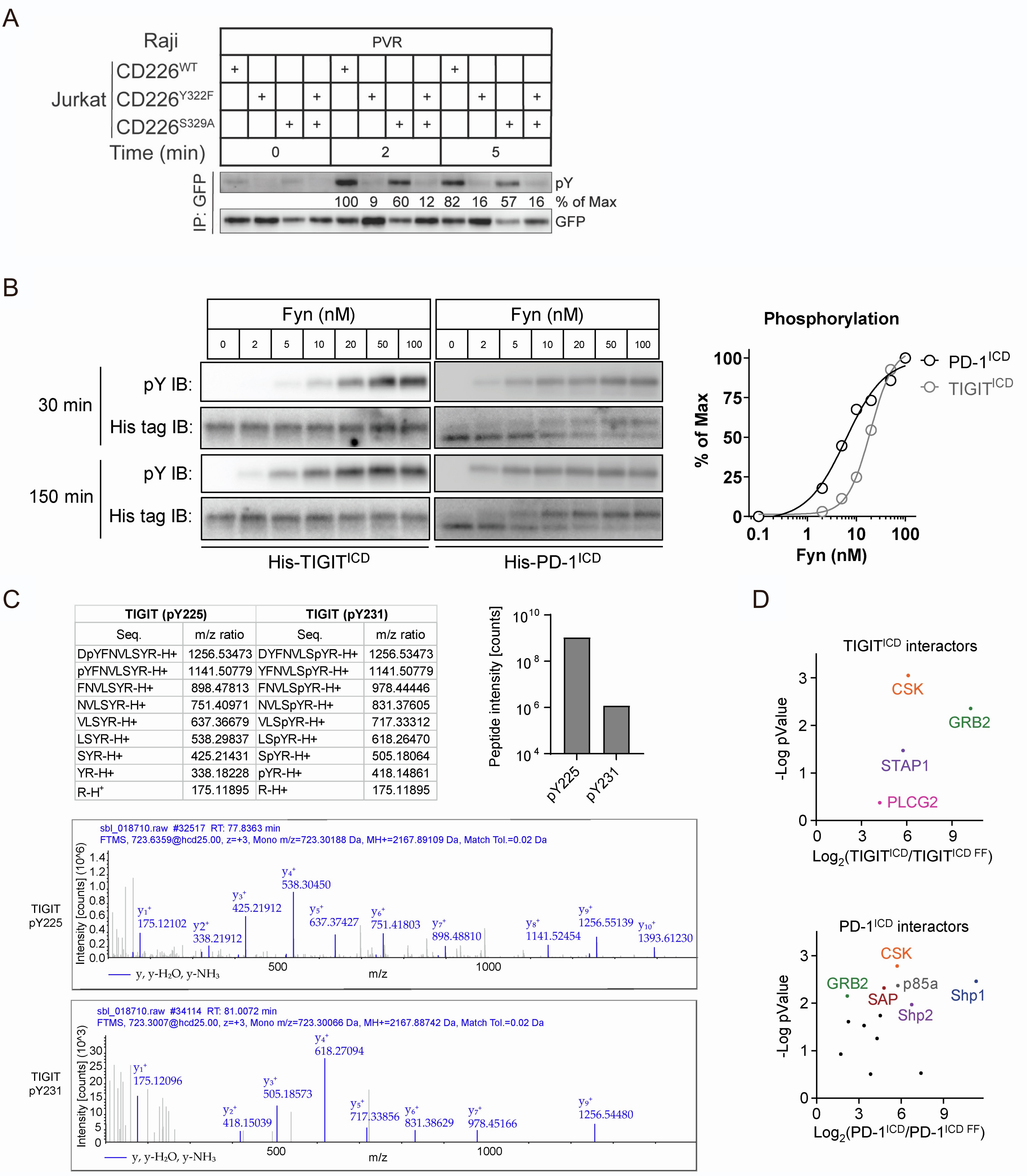

TIGIT (pY225)		TIGIT (pY231)	
Seq.	m/z ratio	Seq.	m/z ratio
DpYFNVLSYR-H+	1256.53473	DYFNVLSpYR-H+	1256.53473
pYFNVLSYR-H+	1141.50779	YFNVLSpYR-H+	1141.50779
FNVLSYR-H+	898.47813	FNVLSpYR-H+	978.44446
NVLSYR-H+	751.40971	NVLSpYR-H+	831.37605
VLSYR-H+	637.36679	VLSpYR-H+	717.33312
LSYR-H+	538.29837	LSpYR-H+	618.26470
SYR-H+	425.21431	SpYR-H+	505.18064
YR-H+	338.18228	pYR-H+	418.14861
R-H+	175.11895	R-H+	175.11895

Figure S4. Preferential phosphorylation of CD226 is at Y322 upon PVR stimulation, and TIGIT^{ICD} tends to be mono-phosphorylated at the ITT motif and failed to recruit Shp1 or Shp2, Related to Figure 5.

Figure S4. Preferential phosphorylation of CD226 is at Y322 upon PVR stimulation, and $TIGIT^{ICD}$ tends to be mono-phosphorylated at the ITT motif and failed to recruit Shp1 or Shp2, Related to Figure 5.

(A) pY IB showing the phosphorylation status of CD226 WT or indicated mutants using after co-culturing Jurkat cells expressing GFP-tagged $CD226^{WT}$, $CD226^{Y322F}$, $CD226^{S329A}$, or $CD226^{Y322F/S329A}$ with SEE-loaded Raji cells expressing PVR. Cells were lysed at the indicated time points after initial contact. "% of max" denotes relative phospho-CD226 levels, normalized to the band with the highest OD. (B) Left, pY IBs showing the phosphorylation of $TIGIT^{ICD}$ and $PD\text{-}1^{ICD}$ as a function of increasing Fyn concentrations. Right, ODs of pY signals, normalized to the highest OD, plotted against Fyn concentration, and fitted with a "Dose-Response-Stimulation" function using Graphpad Prism 8.0. (C) Top left, table summarizing the theoretical m/z (mass/charge) ratios of MSII ions of $TIGIT^{ICD}$ Y225 and Y231 containing peptides. Top right, bar graph showing the intensity (spectral counts) of the most abundant peptide containing pY225 or pY231. Lower, mass spectral data showing the measured m/z ratios of Y225 phosphorylated peptide, and Y231 phosphorylated peptide of $TIGIT^{ICD}$ identified in the GST-$TIGIT^{ICD}$ pull down sample using MS. (D) One-side volcano plot showing MS-identified SH2-containing proteins in GST-$TIGIT^{ICD}$ or GST-$PD\text{-}1^{ICD}$ pull down samples (see Methods). Each dot represents a single SH2-containing protein.

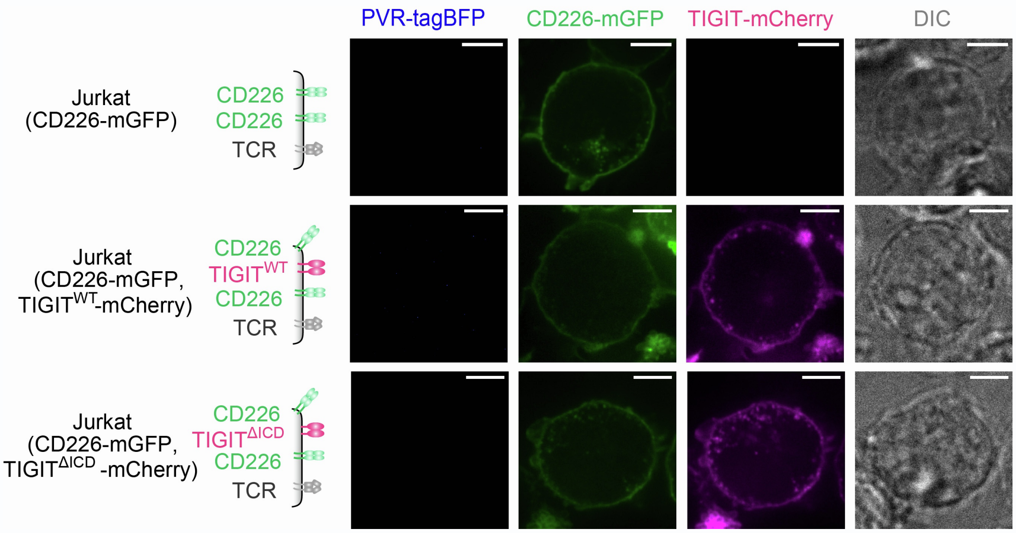

Figure S5. Experimental controls for the Jurkat-Raji cell conjugate assay, Related to Figure 6.

Representative confocal and DIC images of Jurkat T cells expressing CD226-GFP (top row), CD226-GFP with WT TIGIT-mCherry (middle row), or CD226-GFP with TIGIT$^{\Delta ICD}$-mCherry (bottom row) in the absence of Raji cells.

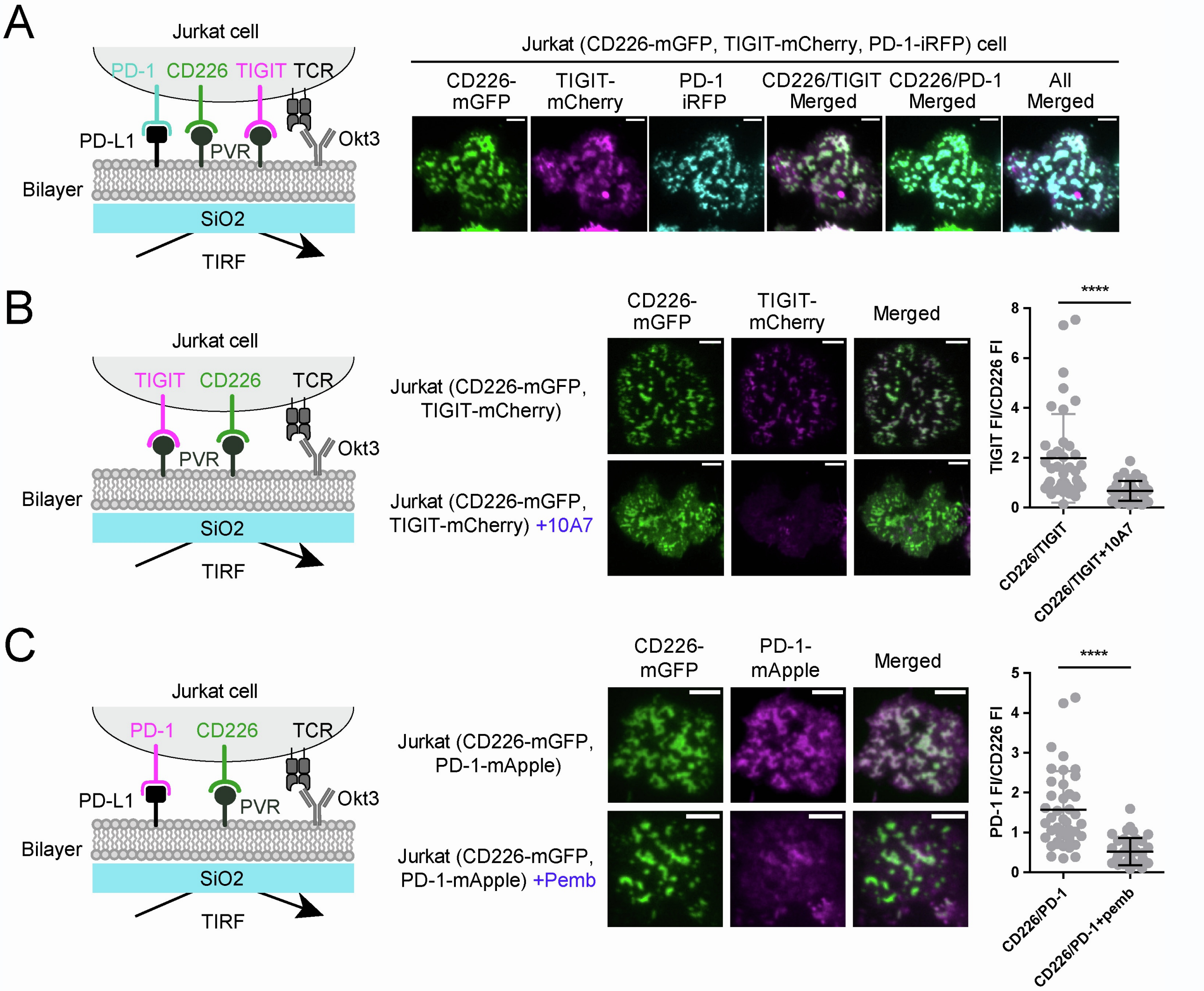

Figure S6. TIGIT and PD-1 both co-localize with CD226 in a ligand-dependent manner, Related to Figure 6.
(A) Left, cartoon depicting a CD226-mGFP/TIGIT-mCherry/PD-1-iRFP triple positive Jurkat cell in contact with an SLB functionalized with anti-CD3ε (clone OKT3), PVR^{ECD} and PD-L1^{ECD}. Right, representative TIRF images showing CD226, TIGIT, and PD-1 microclusters of a single Jurkat cell. (B) Cartoon depicting a CD226-mGFP/TIGIT-mCherry double positive Jurkat cell in contact with an SLB functionalized with anti-CD3ε (clone Okt3) and PVR^{ECD}. Middle, representative TIRF images of CD226 and TIGIT microclusters in a Jurkat cell pretreated by PBS or by a PVR-blocking anti-TIGIT mAb (clone 10A7). Right, dot plots summarizing TIGIT FI-mCherry/CD226-mGFP FI ratios of 40 Jurkat cells under each condition (see Methods). (C) Cartoon depicting a CD226-mGFP/PD-1-mApple double positive Jurkat cell in contact with an SLB functionalized anti-CD3ε (clone Okt3), PVR^{ECD} and PD-L1^{ECD}. Middle, representative TIRF images showing CD226 and PD-1 microclusters in a Jurkat cell pretreated by PBS or by pembrolizumab, a PD-L1 blocking anti-PD-1 mAb. Right, dot plots summarizing PD-1-mApple FI/CD226-mGFP FI ratios of 40 Jurkat cells under each condition (see Methods). Error bars: s.d.. Scale bars: 5 μm. ****$P < 0.001$; Student's t-test.
